# Indirect Costs of Inflammatory Bowel Diseases: A Comparison of Patient-Reported Outcomes Across 12 European Countries

**DOI:** 10.1093/ibd/izac144

**Published:** 2022-07-06

**Authors:** Przemysław Holko, Paweł Kawalec, Magdalena Sajak-Szczerba, Luisa Avedano, Małgorzata Mossakowska

**Affiliations:** Department of Nutrition and Drug Research, Institute of Public Health, Faculty of Health Sciences, Jagiellonian University Medical College, Krakow, Poland; Department of Nutrition and Drug Research, Institute of Public Health, Faculty of Health Sciences, Jagiellonian University Medical College, Krakow, Poland; European Federation of Crohn’s and Ulcerative Colitis Associations, Brussels, Belgium; Polish Association Supporting People with Inflammatory Bowel Disease “J-elita,” Warsaw, Poland; European Federation of Crohn’s and Ulcerative Colitis Associations, Brussels, Belgium; Polish Association Supporting People with Inflammatory Bowel Disease “J-elita,” Warsaw, Poland; International Institute of Molecular and Cell Biology, Warsaw, Poland

**Keywords:** informal care, absenteeism, presenteeism

## Abstract

**Background:**

National studies report a high variability of indirect costs of inflammatory bowel disease (IBD). In this study, selected aspects of the societal burden of IBDs were compared between 12 European countries.

**Methods:**

A questionnaire-based study among adult patients with IBD was performed. Data on patient characteristics, productivity loss, and informal care were collected. The costs of productivity loss were assessed from the social perspective. The cost of absenteeism and presenteeism was valuated using the gross domestic product per worker. Informal care was measured by time inputs of relatives and friends to assist patients. Productivity loss among informal caregivers outside their paid work was valuated with the average wage. The results were adjusted for confounders and multiplicity.

**Results:**

Responses from 3687 patients (67% employed) were analyzed. Regular activity (outside paid work) impairment did not differ between countries, but a significant difference in informal care and productivity loss was observed. There were no differences in indirect costs between the types of IBD across the countries. The mean annual cost of absenteeism, presenteeism, and informal care varied from €1253 (Bulgaria) to €7915 (Spain), from €2149 (Bulgaria) to €14 524 (Belgium), and from €1729 (Poland) to €12 063 (Italy), respectively. Compared with patients with active disease, those with IBD in remission showed a lower indirect cost by 54% (presenteeism, *P <* .001) or 75% (absenteeism, informal care, *P <* .001).

**Conclusions:**

The study showed a high relevance of the indirect cost of IBD in the context of economic evaluation, as well as a between-country variability of work-related impairment or informal care.

**Key Messages**

What is already known?

- There are a limited number of national studies assessing indirect costs of inflammatory bowel disease (IBD).

What is new here?

- Regular activity impairment of IBD patients did not differ between countries.
- The difference in work-related impairment of patients with IBD from different countries was significant, especially after consideration of macroeconomic factors for each country.
- After controlling for disease severity and other confounders, no differences in indirect costs were observed between Crohn’s disease patients and ulcerative colitis patients.

How can this study help patient care?

- Future studies should consider variability between countries in work-related impairment or informal care.

## Introduction

Productivity costs usually include the loss of productivity at paid and unpaid work of patients and their caregivers, as assessed from the societal perspective. Indirect costs of an illness involve mainly temporary absence from paid work (absenteeism), lower-than-average workforce productivity during paid work (presenteeism), permanent work disability or unemployment, loss of the patient’s productivity at unpaid work (household work, voluntary work), loss of leisure time, and time inputs of relatives and friends into the care of the patient (informal care).^1,2^

Inflammatory bowel diseases (IBDs), which primarily include Crohn’s disease (CD) and ulcerative colitis (UC), affect people of all ages and constitute a significant burden for the patient and the society.^3,4^ Because of an early onset and chronic nature, IBDs affect work productivity, with productivity loss resulting from sick leave and work disability amounting up to 50% of the total cost of the disease.^4–6^ A high burden in work-related outcomes among patients with IBD has been documented, although only in a limited number of national studies assessing productivity loss at paid work due to IBDs.^4,7^ A recent systematic review^4^ indicated a high variability of indirect costs in relation to world regions, IBD type, and patient population, with the broadest range of productivity costs observed in Europe (from $640 to $6000 per patient per year). Only a few studies assessing productivity costs were identified, and most of them reported on absenteeism only. They were also limited by their focus on a single country, which makes any comparisons between them difficult. Moreover, a meta-analysis by Kawalec and Malinowski^7^ indicated a high heterogeneity of available information on productivity costs among patients with IBD, which cannot be overcome by adjustments for macroeconomic indicators. The cost of absenteeism and early departure from the labor market due to IBD varied worldwide from $515 to $14 727 per patient per year.^7^

To date, only 2 studies have been identified to report the cost of informal care in IBDs. They suggested a considerable difference in the cost between IBD types.^4^ The inclusion of informal care can have a strong impact on cost-effectiveness outcomes. It has been recommended to consider the relevance of informal care in the context of economic analysis (eg, test an association with disease activity) and to include it or justify its exclusion.^8^ However, limited information on the informal care of patients with IBD precludes either.

Data on the indirect costs of IBD are lacking for some European countries, and it is unclear whether different estimates in national studies are due to between-country differences in patient populations, methods, or healthcare, occupational, and social care systems. Lönnfors et al^9^ carried out a survey among 4670 patients with IBD from 25 European countries and revealed a high impact of IBD on patient productivity, but the study was not designed to address potential between-country differences. Therefore, the present study aimed to assess the selected aspects of the societal burden of IBDs and compare them between European countries. Another objective was to check those outcomes in relation to disease activity, IBD types, and other patient characteristics. The study was conducted in collaboration with the European Federation of Crohn’s and Ulcerative Colitis Associations (EFCCA).

## Methods

### Study Design

This was a multinational online questionnaire study. Subjects 18 years of age or older with a diagnosis of IBD were invited by national patient associations allied within the EFCCA. Several dissemination techniques of information about the study were implemented by national patient associations and EFCCA (direct emails to members, at forums and Web portals, at events organized by associations, in their publications for patients). The only restriction of the study was access to the Internet and the ability to complete the Web application. However, patients without computer skills were encouraged to use the help of relatives or representatives of patient associations.

No patient identifiable information was collected, and none of the questions were obligatory. Participation in the study was voluntary and anonymous. The information about the study was available to all patients prior to participation. The collection of responses was initialized only after obtaining the patient’s consent.

The questionnaire included questions relating to (1) general information about the respondent (current age, age at diagnosis, sex, comorbidities, and place of residence [respondents selected 1 of the following options: a city with a population of ≥100 000; a city with a population of <100 000; or a village]); (2) the patient Harvey-Bradshaw Index (P-HBI)^10^ or Patient Simple Clinical Colitis Activity Index (P-SCCAI) score^11^; (3) disease activity as assessed by a specialist during the last consultation and the time from this consultation (used to verify the activity status based on the P-HBI or P-SCCAI score); (4) previous surgical treatment of IBD and the time from the last procedure; (5) current pharmacotherapy of IBD; (6) occupational status; (7) monthly out-of-pocket patient expenses; (8) work productivity and regular activity impairment in the past 7 days, using the Work Productivity and Activity Impairment Questionnaire (WPAI)^12^; and (9) informal care (patients were asked to indicate the number of hours that the family members and unpaid nonrelatives dedicated to IBD-related care in the past 7 days). The questionnaires are provided in the Supplementary Appendix.

The study procedures were approved by the representatives of national patient associations allied within the EFCCA and gastroenterologists, whose comments were collected and included in the final version of the questionnaires. The WPAI is available in various languages. However, other questions and the general study information for participants were prepared in English and translated by the representatives of patient associations. They were then translated back to check for potential errors.

The study was conducted from October 2018 to October 2019.

### Data Management

Responses to the P-HBI or P-SCCAI were analyzed according to the instructions provided by Bennebroek Evertsz’ et al (P-HBI score >4 and P-SCCAI score >5 denoting active disease).^10,11^ The social perspective was adopted, and the human capital approach was used to estimate productivity cost at paid work. The healthy time lost due to the disease was valuated using gross domestic product per working hour of a person with occupational activity in a country in 2019.^13,14^ This approach can be perceived as the loss of an investment in a person’s human capital. However, recognizing that only part of the population has an occupational activity, working time is not the only factor of production, and reduced performance or absence of an employee also prevents the use of complementary factors of production (quality of the workforce). Additionally, the conventional mean value of output elasticity of labor according to the Cobb-Douglas function of production (0.65) was implemented to adjust the unit cost according to the law of diminishing marginal productivity.^15^ The productivity loss among informal caregivers (informal care) outside their paid work was measured with the inclusion of the time spent on the care of a participant, using the recall method.^16^ The opportunity cost method^17^ was used to value informal care with unit cost at an average wage per hour of work in a country in 2019.^18^ Unit costs are presented in Table 1 and Supplementary Table 1.

**Table 1.** Unit Cost of Productivity Impairment

	Unit Cost (Hour) of Work Lost (in 2019 €)	Unit Cost (Hour) of Time Lost Outside Work (in 2019 €)
Belgium	40.26	39.90
Bulgaria	6.88	6.10
Cyprus	18.57	14.80
Czech Republic	15.12	12.30
Denmark	49.00	41.70
Greece	12.56	10.20
Hungary	11.70	8.30
Italy	26.75	23.40
Poland	10.59	8.10
Portugal	14.87	12.10
Romania	9.30	6.90
Spain	23.60	20.50

The indirect cost was calculated as a product of the time of productivity loss due to IBD during the previous week (ie, hours missed from paid work, hours missed due to reduced quality of production during paid work, hours of informal care) and unit costs. The weekly estimates were then annualized. All cost outcomes were presented in 2019 euros (the period to which cost data applied).

### Statistical Analysis

All study outcomes and patient characteristics were presented as mean ± SD for continuous variables and as frequencies for categorical variables. Correlations were assessed by the Spearman’s ρ rank correlation coefficient. The Pearson chi-square test for categorical variables and the Kruskal-Wallis rank test for continuous variables were applied for comparison between the subgroups.

The fractional logit regression was used to analyze regular activity impairment, absenteeism, and presenteeism scores (WPAI). Two-part models (*twopm*)^19^ were used to analyze indirect costs.^20^ The first stage of the 2-part models refers to whether the cost is positive (ie, nonzero), while the second stage refers to its amount, conditional on the cost being positive. The models were fitted with a robust estimator of variances and included a categorical variable for IBD type, current disease activity (remission or active disease assessed with the P-HBI or P-SCCAI score), and country, and covariates to control for possible confounders. Interactions between variables were included if relevant. Model selection and assessment were based on the Box-Cox test, modified Park test, and pseudo-log-likelihood. Average adjusted predictions and average marginal effects were presented as adjusted means and differences.

The analyses included all questionnaires with at least 1 answer. Missing data were excluded from the analysis of an outcome. The Bonferroni correction for multiple hypothesis testing was incorporated. To ensure self-explanatory attribute of the results, the *P* values and confidence intervals (CIs) were adjusted with the correction, that is, the adjusted *P* values were presented as *P* values and CIs adjusted for multiplicity were presented as 95% CIs. The adjusted *P* value of <.05 (nominal *P* value of <.00025) was considered statistically significant.

A country was included in the study if there were at least 50 patients with IBD from that country who provided an answer to at least 1 question from the questionnaires. A priori power analysis was not performed, but the minimum sample size from a country was derived using a power of 80%, the level of significance of 0.01%, and the ability to detect a 20% difference in the indirect cost between any 2 independent samples from separate countries assuming equal variances, 10% of missing responses, and an equal recruitment ratio.

The study was reported in adherence with the STROBE (Strengthening the Reporting of Observational Studies in Epidemiology Statement) recommendations.^21^ Data preparation and statistical analyses were done using STATA 17SE (StataCorp, College Station, TX, USA).

## Results

The questionnaires from 3687 respondents representing 12 countries were collected (Table 2). Reported characteristics did not differ between complete cases and patients with missing answers. There were significant differences between countries in most patient characteristics, except for the prevalence of some comorbidities and treatments (ciclosporin, golimumab, certolizumab), mean P-SCCAI score among patients with UC, current activity status of UC, prevalence of penetrating CD course, and the proportion of students (Supplementary Table 2). There were 2455 (67.0%) working patients (from 48.9% among patients from Greece to 75.0% among those from Hungary; *P <* .001). Among working patients, 1191 had UC (894 in remission; *P =* .081 between countries) and 1259 had CD or other IBDs (604 in remission; *P =* .038) (Supplementary Table 3).

**Table 2.** Characteristics of Study Participants

	All Patients (N = 3687)	Patients With Occupational Activity (n = 2455)
Age, y	43.03 ± 13.76	41.40 ± 10.91
Male	1241 (34.0)	848 (34.9)
Age at diagnosis, y	29.81 ± 12.42	28.94 ± 10.61
Disease		
CD	1930 (52.4)	1230 (50.1)
UC	1693 (45.9)	1194 (48.6)
Other IBD	63 (1.7)	31 (1.3)
Country		
Belgium	128 (3.47)	73 (2.97)
Bulgaria	141 (3.82)	104 (4.24)
Cyprus	53 (1.44)	32 (1.30)
Czech Republic	69 (1.87)	48 (1.96)
Denmark	1253 (33.98)	833 (33.93)
Greece	264 (7.16)	127 (5.17)
Hungary	77 (2.09)	57 (2.32)
Italy	196 (5.32)	115 (4.68)
Poland	467 (12.67)	349 (14.22)
Portugal	651 (17.66)	472 (19.23)
Romania	131 (3.55)	74 (3.01)
Spain	257 (6.97)	171 (6.97)
With comorbidities	1893 (56.1)	1173 (52.9)
Current pharmacotherapy		
Sulfasalazine	416 (11.5)	291 (12.0)
Mesalazine	1537 (42.5)	1067 (44.1)
Plain steroids	653 (18.0)	403 (16.7)
Budesonide	255 (7.0)	169 (7.0)
Azathioprine	966 (26.7)	658 (27.2)
Mercaptopurine	96 (2.7)	66 (2.7)
Methotrexate	143 (4.0)	80 (3.3)
Adalimumab	357 (9.9)	226 (9.3)
Infliximab	569 (15.7)	391 (16.2)
Golimumab	24 (0.7)	15 (0.6)
Vedolizumab	151 (4.2)	100 (4.1)
Ciclosporin	21 (0.6)	13 (0.5)
Metronidazole	147 (4.1)	87 (3.6)
Beclomethasone	19 (0.5)	12 (0.5)
Certolizumab	5 (0.1)	4 (0.2)
Ustekinumab	77 (2.1)	46 (1.9)
Past surgical treatment		
Previous year	268 (7.3)	144 (5.9)
1-5 y ago	461 (12.6)	299 (12.2)
5+ y ago	580 (15.8)	367 (15.0)
Current disease severity		
Remission	2160 (58.9)	1498 (61.1)
Active disease	1509 (41.1)	952 (38.9)
UC patients with stoma	132 (7.8)	87 (7.3)
Penetrating CD course	595 (30.9)	376 (30.7)
Retired	380 (10.3)	140 (5.7)
On a disability pension	384 (10.4)	177 (7.2)
With disability certificate	523 (14.2)	90 (3.7)
Student	312 (8.5)	14 (0.6)
Registered unemployment	180 (4.9)	—
Not registered unemployment	160 (4.3)	—
During short-term absence from work	407 (11.0)	—

Values are mean ± SD or n (%).

Abbreviations: CD, Crohn’s disease; IBD, inflammatory bowel disease; UC, ulcerative colitis.

There was no significant correlation between country and IBD type or disease activity, but a weak association between disease activity and IBD type (ie, a higher proportion of active CD) was observed (ρ = -0.28, *P <* .001).

### Regular Activity Impairment

**Figure 1.**
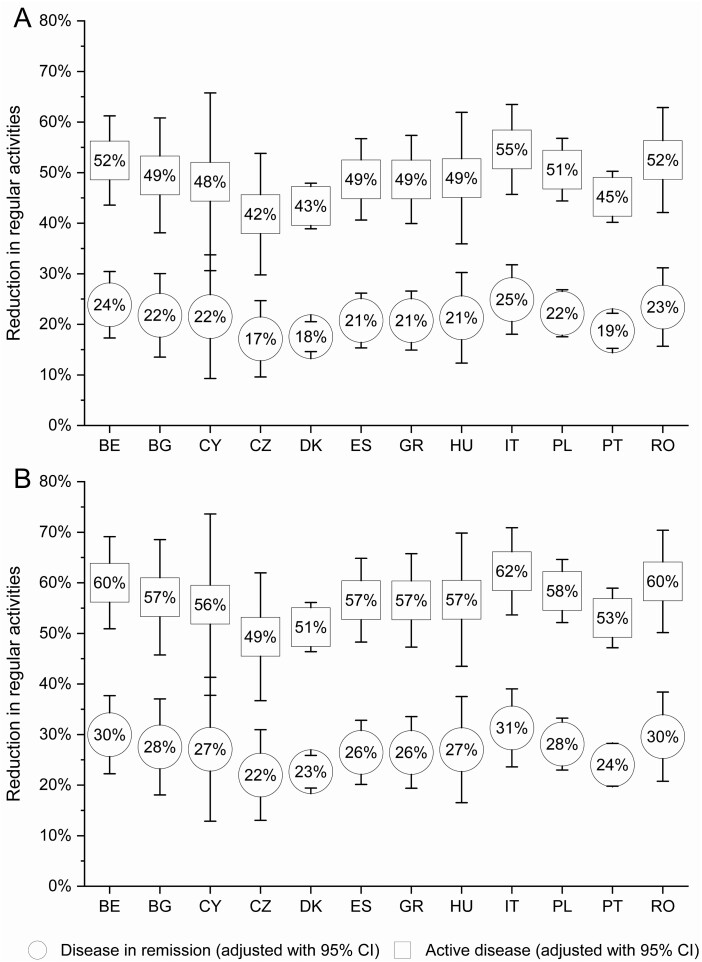
Adjusted mean of regular activity impairment among patients with A, Crohn’s disease; and B, ulcerative colitis by country. BE, Belgium; BG, Bulgaria; CI, confidence interval; CY, Cyprus; CZ, Czech Republic; DK, Denmark; ES, Spain; GR, Greece, HU, Hungary; IT, Italy; PL, Poland; PT, Portugal; RO, Romania.

More than three-fourths of the participants indicated a reduction of their regular activities due to IBD (62.5% among patients with disease in remission and 92.9% among those with active disease). There was no significant difference between different countries in terms of the reduction of regular activity impairment (Figure 1). Younger patients (odds ratio [OR], 0.99 per year; *P =* .006), men (OR, 0.83; *P =* .009), patients with UC (OR, 1.38; *P <* .001), patients with active disease (OR, 3.70; *P <* .001), patients who had undergone surgery in the past year (OR, 1.48; *P <* .001), patients with comorbidities (OR, 1.31; *P <* .001), and patients requiring biological treatment (OR, 1.21; *P =* .014) experienced a greater reduction of regular activity impairment (Supplementary Table 4).

### Informal Care

Around 32% of the patients (21% of those with disease in remission and 48.6% of those with active disease) reported informal care due to IBD. The proportion of respondents reporting assistance differed between countries, from 20.5% in Denmark to 64.1% in Romania (*P <* .001). Family members and nonrelatives dedicated a mean of 5.1 ± 22.44 hours per week, including 1.7 ± 11.12 hours leaving their paid work. The mean cost of informal care was estimated at €4468 ± 18 055.36 per patient per year. Patient age (*P <* .001), presence of comorbidities (38% increase; *P =* .025), occupational activity (53% reduction among working patients; *P <* .001), disease activity (75% reduction if disease in remission; *P <* .001), and country (*P <* .001) were significantly associated with informal care cost (Supplementary Table 5). Patients with active disease, patients with comorbidities, and patients from different countries showed higher odds of having nonzero cost of informal care (ie, >€0; first part of the 2-part model), and had a higher cost among patients with nonzero cost of informal care (second part of the 2-part model). However, younger patients and nonworking patients had higher odds of having nonzero cost only. Among patients reporting informal care, the cost did not differ in relation to age or occupational status. There was no difference in the cost of informal care between IBD types (UC vs CD: -€530 per year per patient, 95% CI, -€2429 to €1369), but patients with disease in remission had a lower adjusted cost of informal care by 75% in relation to those with active disease (the difference of €5368 per year per patient, 95% CI, €3336 to €7400; *P <* .001). A significant variability between countries in the amount of impact of IBD activity was observed. The marginal cost ranged from €2210 among UC patients from Poland to €14 880 among CD patients from Italy. The overall cost of informal care was the lowest among patients from Poland, Czech Republic, and Portugal and the highest among patients from Italy and Spain (Table 3).

**Table 3.** The Work-Related Indirect Costs (per Working Patient per Year) and Informal Care Cost (per Patient per Year) by Country, Disease Type, and Disease Activity

Country	IBD	Informal Care (in 2019 €)			Absenteeism (in 2019 €)			Presenteeism (in 2019 €)		
		Remission (SE)	Active Disease (SE)	Difference (SE)	Remission (SE)	Active Disease (SE)	Difference (SE)	Remission (SE)	Active Disease (SE)	Difference (SE)
Belgium	CD	2579 (640)	9488 (1798)	6908 (1308)	2541 (867)	10 479 (2667)	7939 (1959)	13 538 (1733)	24 956 (2180)	11 418 (1238)
	UC	2277 (578)	8325 (1737)	6048 (1285)	3323 (1087)	12 065 (3060)	8742 (2193)	16 405 (2033)	29 862 (2710)	13 458 (1567)
Bulgaria	CD	2849 (857)	9766 (2602)	6916 (1870)	800 (282)	3377 (965)	2576 (727)	2143 (346)	4846 (561)	2703 (321)
	UC	2513 (744)	8558 (2323)	6046 (1686)	1051 (357)	3910 (1114)	2859 (815)	2638 (409)	5852 (681)	3213 (396)
Cyprus	CD	2328 (1267)	10 854 (5236)	8526 (4036)	1298 (597)	5446 (1861)	4147 (1340)	4237 (926)	8327 (1277)	4090 (644)
	UC	2062 (1149)	9567 (4794)	7505 (3705)	1704 (765)	6296 (2147)	4593 (1498)	5162 (1101)	9991 (1562)	4829 (799)
Czech Republic	CD	954 (299)	3783 (917)	2829 (668)	2420 (1020)	9502 (3187)	7081 (2298)	2284 (592)	5869 (1001)	3586 (525)
	UC	843 (262)	3324 (830)	2480 (612)	3135 (1262)	10 809 (3540)	7674 (2461)	2836 (712)	7135 (1207)	4300 (641)
Denmark	CD	1766 (351)	8210 (1328)	6444 (1087)	3005 (590)	15 333 (2474)	12 327 (2125)	9740 (678)	21 459 (1096)	11 719 (933)
	UC	1565 (285)	7236 (1157)	5671 (970)	4087 (680)	18 749 (2750)	14 662 (2401)	11 969 (759)	25 880 (1480)	13 911 (1217)
Greece	CD	1576 (335)	5745 (1013)	9650 (2205)	935 (260)	3471 (788)	13 326 (2868)	4066 (549)	8523 (845)	6707 (717)
	UC	1391 (303)	5040 (975)	8474 (2086)	1197 (314)	3897 (865)	14 462 (2955)	4978 (654)	10 255 (1051)	8024 (889)
Hungary	CD	1112 (834)	4989 (3602)	2519 (390)	3973 (3277)	17 074 (13 426)	4160 (1229)	3543 (497)	6993 (670)	3817 (385)
	UC	985 (741)	4394 (3196)	2210 (370)	5239 (4278)	19 865 (15 524)	4745 (1183)	4318 (604)	8392 (866)	4506 (474)
Italy	CD	5402 (1354)	20 282 (4432)	3622 (560)	4432 (1184)	16 531 (3266)	4814 (740)	6770 (992)	15 120 (1522)	5453 (543)
	UC	4771 (1192)	17 802 (4077)	3183 (563)	5679 (1404)	18 584 (3524)	5258 (896)	8328 (1173)	18 247 (1865)	6477 (697)
Poland	CD	828 (151)	3347 (484)	5562 (1086)	1166 (366)	5325 (1539)	4081 (932)	3986 (350)	7803 (571)	3185 (413)
	UC	732 (127)	2942 (450)	4850 (992)	1555 (395)	6300 (1495)	4335 (1072)	4854 (381)	9360 (682)	3764 (509)
Portugal	CD	1089 (195)	4711 (679)	3877 (2789)	1592 (289)	6406 (890)	13 101 (10 249)	4439 (399)	9892 (744)	3450 (390)
	UC	963 (178)	4146 (681)	3409 (2474)	2072 (368)	7330 (1102)	14 626 (11 385)	5460 (483)	11 937 (979)	4074 (505)
Romania	CD	2753 (595)	8315 (1511)	14 880 (3351)	1523 (461)	5604 (1280)	12 099 (2394)	3170 (507)	6355 (757)	8350 (891)
	UC	2422 (503)	7272 (1349)	13 032 (3115)	1946 (575)	6281 (1504)	12 905 (2538)	3868 (596)	7632 (918)	9919 (1112)
Spain	CD	2978 (796)	12 627 (2865)	4169 (771)	4528 (1154)	17 854 (3722)	2535 (590)	4520 (666)	11 227 (1158)	4457 (496)
	UC	2634 (712)	11 109 (2684)	3649 (748)	5869 (1335)	20 331 (3877)	2700 (632)	5600 (776)	13 624 (1407)	5276 (624)

Abbreviations: CD, Crohn’s disease; IBD, inflammatory bowel disease; UC, ulcerative colitis.

### Absenteeism and Presenteeism

**Figure 2.**
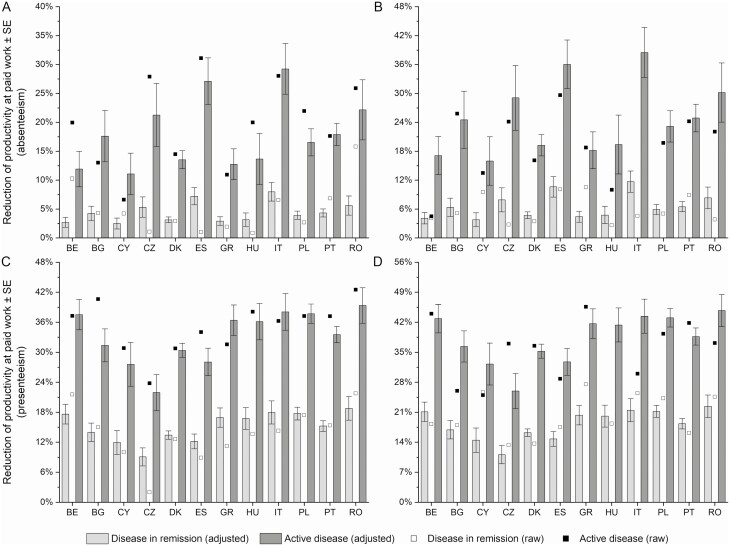
Impairment of productivity at paid work among study participants with occupational activity by disease activity and country: A, absenteeism score among patients with Crohn’s disease; B, absenteeism score among patients with ulcerative colitis; C, presenteeism score among patients with Crohn’s disease; and D, presenteeism score among patients with ulcerative colitis. BE, Belgium; BG, Bulgaria; CY, Cyprus; CZ, Czech Republic; DK, Denmark; ES, Spain; GR, Greece, HU, Hungary; IT, Italy; PL, Poland; PT, Portugal; RO, Romania.

The productivity at paid work was strongly impaired among study participants. Working patients missed a mean of 3.38 ± 21.71 hours of paid work per week (absenteeism). Patients had a reduced quality of production during paid work (presenteeism), resulting in a mean loss of 5.27 ± 9.45 working hours per week. There was a moderate variability in absenteeism and presenteeism scores between countries that reached borderline significance (*P =* .052 and *P =* .040, respectively) (Figure 2). Among tested variables, disease activity showed a strong association with absenteeism (OR, 1.54) and presenteeism (OR, 1.25) scores (both *P <* .001). Additionally, patients who required biological treatment (OR, 1.53; *P =* .152) and those with surgery in the past year (OR, 2.29; *P =* .007) had a higher absenteeism score. On the other hand, patients with comorbidities had a higher presenteeism score (OR, 1.31; *P =* .002) (Supplementary Table 4).

The mean cost of absenteeism and presenteeism was estimated at €3782 ± 17 644.50 and €6457 ± 12 146.15 per working patient per year, respectively. Overall, the cost of absenteeism and presenteeism differed significantly between countries (*P <* .001) and disease activity (*P <* .001) but not between IBD types (UC vs CD: €1172 [95% CI, -€2169 to €4512] for the absenteeism cost and €1942 [95% CI, -€176 to €4061] for the presenteeism cost; Table 3). Patients with active disease had higher absenteeism and presenteeism costs by €8585 (95% CI, €4868 to €12 302) and €8063 (95% CI, €5715 to €10 411) per working participant per year, respectively. A significant variability between countries in the amount of impact of IBD activity was observed. The marginal cost ranged from €2535 among CD working patients from Greece to €14 662 among working UC patients from Denmark (absenteeism), and from €2703 among working CD patients from Bulgaria to €13 911 among working UC patients from Denmark (presenteeism).

Patients with active disease had both higher odds of having a nonzero cost (*P <* .001; the first stage of the 2-part model) and a higher cost among patients with a nonzero cost (absenteeism, *P =* .016; presenteeism, *P <* .001; the second stage of the 2-part model). However, the difference between countries was not significant in the first part of the 2-part models, which means that the odds of having a nonzero cost was not different between countries. Patients from different countries differed in the amount of the cost only.

The absenteeism cost was the lowest among patients from Bulgaria, Greece, Poland, and Cyprus and the highest among patients from Italy, Spain, Denmark, and Hungary. The presenteeism cost was the lowest in Bulgaria and Czech Republic and the highest in Denmark and Belgium. Among patients with any cost, those with UC had a higher cost of presenteeism (*P =* .011) than patients with other IBDs (Supplementary Table 5). The adjusted overall difference in the presenteeism and absenteeism cost between IBD types was €1942 (95% CI, -€176 to €4061) and €1172 (95% CI, -€2169 to €4512), respectively. Additionally, patients who required biological treatment (47% increase; *P =* .028) and patients with surgery in the past year (69%-115% increase vs other patients; *P =* .493) had a higher absenteeism cost, while patients with comorbidities had a higher presenteeism cost (14% increase; *P =* .012).

**Figure 3.**
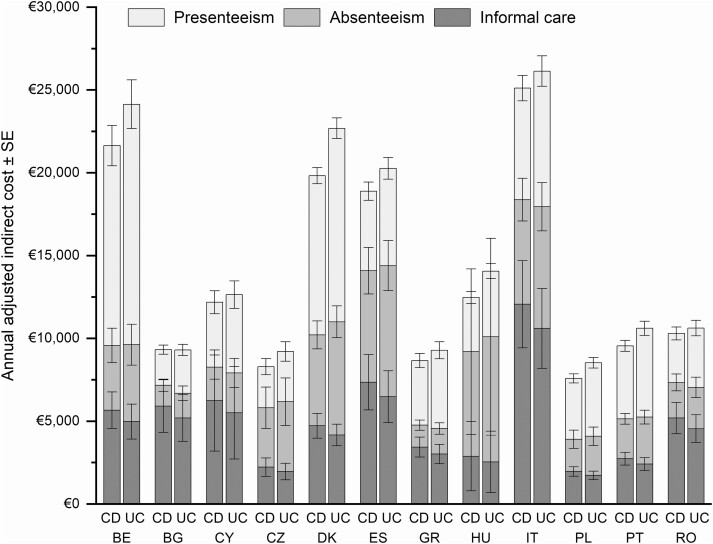
Adjusted indirect costs per patient per year by country and the type of inflammatory bowel disease (Crohn’s disease [CD] or ulcerative colitis [UC]). BE, Belgium; BG, Bulgaria; CY, Cyprus; CZ, Czech Republic; DK, Denmark; ES, Spain; GR, Greece, HU, Hungary; IT, Italy; PL, Poland; PT, Portugal; RO, Romania.

Raw estimates of indirect costs by country, disease activity, and IBD type are presented in Supplementary Table 6, while adjusted mean indirect costs by country and IBD type are presented in Supplementary Table 7. The adjusted mean annual cost of absenteeism, presenteeism, and informal care ranged from €7583 (CD, Poland) to €26 143 (UC, Italy) (Figure 3).

## Discussion

To our best knowledge, this is the first study reporting an indirect cost of IBD exclusively among patients from Portugal, Greece, Cyprus, and Bulgaria. The strengths of the study include the sample size, multinational background of the respondents, and diversity of the participants, achieved by direct enrollment without additional restrictions (eg, during consultation with a specialist or hospitalization). The limitations include the fact that self-reported information can be influenced by recall, response, or social desirability biases. The disease activity assessment using P-HBI or P-SSCI was another limitation because the method does not ensure the perfect agreement with the clinician assessment.^10,11^ However, the comparison between current disease activity and disease activity assessed by a clinician during the last consultation revealed a moderate agreement (65.1%, Cohen’s κ of 0.32), with more than two-thirds of patients not consulting their disease status in a month preceding enrollment and around 30% having their consultations more than 3 months before the study. We observed that the longer the time from the clinician’s assessment was, the higher the probability of disagreement was. This indirectly confirms the validity of the patient questionnaires.

Our study sample was not necessarily representative of the IBD population as a whole, and the sample size differed between countries. It was not possible to ensure that patients from each country represented its whole population, and there were no significant interactions between a country and disease severity or its activity. Consequently, we could not assume that the difference between countries in the share of patients with UC or with active disease, for example, was representative of the whole population in those countries. Therefore, we presented the average adjusted estimates of the study outcomes, which borrows the strength of the total sample size in the calculation of the national values using the characteristics of patients from the whole sample. The characteristics of UC patients in our study were mostly comparable with those of the physician-reported patient population included in the European LUCID (ie, living with UC; identifying the socioeconomic burden in Europe) study.^22^ However, we observed a higher share of patients with UC in remission, which might be attributed to different enrollment procedures in our study. The lack of representativeness of the sample from a country does not necessarily undermine our conclusions, as the assessment of the variability between countries in the results was adjusted for potential differences in known patient characteristics, disease characteristics, and treatments that they received. However, this issue could affect the average predictions for each country and group of patients.

We did not measure the causal relationship between disease activity and indirect costs, but our results suggest that the induction and maintenance of remission in participants with active disease might reduce the indirect cost of IBD by 54% (presenteeism) and 75% (informal care, absenteeism), thus providing an argument to highly prioritize effective IBD treatments, even under restricted budgetary conditions. Nevertheless, because data were collected once for each participant, the relationship between the outcomes and disease activity requires additional confirmation in a longitudinal study.

We found only moderate variability in nonmonetary work–related outcomes between countries, which may be the result of similar societal and healthcare policies (eg, sick leave payment, universal health insurance). We did not measure specific aspects that may be responsible for the differences between countries. The observed difference in absenteeism and presenteeism scores may be cultural or may be caused by differences in labor policies. Lifestyle factors or socioeconomic situations that were not evenly distributed between countries may also be responsible, but those aspects were not measured in our study. Some differences in healthcare systems can also be responsible. For example, the frequency of hospitalizations among patients with IBD was shown to vary significantly between countries,^23^ and the same medical procedure can be performed in a hospital or outpatient setting depending on the health system.

A systematic review by Leso et al^24^ identified disease severity and comorbidity as predictive factors of work disability among patients with IBD. Conflicting evidence of higher temporary work-related activity impairment among patients with CD vs those with UC was found. We confirmed that the presence of comorbidities, more severe disease course (current active disease, need for biological treatment, or surgery in the past year), and also the country of residence had the strongest association with work-related activity impairment and indirect costs after controlling for known confounders. The work-related activity impairment did not differ significantly between IBD types, but a significant correlation between disease activity and IBD type was observed. This suggests that the difference in work-related impairment between CD and UC observed in some cross-sectional or cohort studies resulted from higher disease severity among CD patients than among those with UC (eg, a higher proportion of patients with severe disease).^24^ In a recent study of 510 patients from the Netherlands, van Gennep et al^25^ identified disease activity and active perianal disease as the only predictors of work productivity loss in IBD. In line with our findings, they found that work-related activity impairment did not differ between IBD types. Similarly, Shafer et al^26^ did not find a significant difference between the indirect cost of CD and UC in their large registry-based study of 849 patients from Canada but showed a high correlation between the indirect costs of IBD and the IBD disability index.

In the LUCID study,^22^ the indirect costs of UC in 1657 patients from 10 countries (Denmark, France, Germany, Italy, Norway, Poland, Spain, Turkey, United Kingdom, and Romania) were analyzed. The only indirect-cost categories were as follows: nonprofessional caregiving, retire or stop working, and time off work in the last 12 months (absenteeism). The study revealed significant differences in indirect costs between patients with moderate-to-severe UC and UC in remission or mild UC (patients with mild UC, 54%). The mean annual indirect cost for all countries was €3098 among patients with moderate-to-severe UC and €2309 among those with mild UC or UC in remission. The overall indirect costs differed between countries. The highest mean indirect cost was recorded for patients from France (€4334), followed by Spain (€3061), the United Kingdom (€3045), and Italy (€2876). The lowest indirect cost was reported for patients from Norway (€0), Turkey (€80), and Romania (€820). In the LUCID study, the indirect cost was significantly lower than that in our analysis, and the difference between activity groups was smaller. However, the LUCID study did not adjust for confounders between UC activity groups and between countries, which may have affected the comparisons. Moreover, the authors used a nonstandardized questionnaire to assess the cost (eg, included time missed from work during the last 12 months for each participant) and applied a unit cost based on average salaries in the different countries (lower than in our study). Finally, according to the LUCID investigators, the fluctuating nature of UC may have affected the results. For example, during the period of indirect cost assessment (last year), UC severity may have changed several times. In our study, we assessed current disease activity and indirect costs during the previous week (and the values were then annualized). We also included the presenteeism cost, which amounted to around 50% of the total indirect cost in our study.

Armuzzi et al^27^ observed high impact of UC activity on impairment in regular and work-related activity among UC patients from 5 European countries. The difference of absenteeism and presenteeism scores between active disease and disease in remission (absenteeism: 29% vs 3%-4%; presenteeism: 49% vs 11%-16%) reported by Armuzzi et al is in line with our results (Figure 2). The authors did not focus on between-country differences, but the reported values of regression model coefficients suggest that patients from Italy had higher regular and work-related activity impairment than those from Spain.^27^ Numerically higher impairment scores among patients from Italy than among those from Spain were observed in our study, but the difference in regular activity impairment was not significant after controlling for known confounders. However, the difference in work-related impairment between countries was statistically significant, especially after consideration of macroeconomic factors for each country (ie, number of working hours and gross domestic product for each country to value the impairment).

Our results are in line with previous studies including patients from the analyzed countries. In the only studies that assessed the informal care cost of IBD, as identified by van Linschoten et al,^4^ the annual cost was estimated at around €579 among 195 CD patients and €1001 among 147 UC patients from Poland.^28,29^ These estimates are lower than those in the present study, but they were calculated using a unit cost of informal care nearly 2 times lower than that used in the present study (€5.29 and €5.90).^28,29^ In both of those studies, the difference of informal care cost between remission and active disease groups was almost the same as in the present study (75% reduction). The absenteeism and presenteeism scores were also comparable, but higher impairment was reported for patients with active UC. In the CD study,^28^ a reduction of an indirect cost in the remission and active disease groups was comparable to our current findings. As for the UC study,^29^ we noted a difference only for the reduction of the presenteeism cost in the remission and active disease groups (15% vs 54% in the present study).^29^ The proportions of patients reporting assistance and of those with active disease were also in line with our dataset. However, both those studies included a lower proportion of patients using biological treatment (14% in the study by Holko et al and 13% in the study by Kawalec et al vs 31% of patients overall in the present study).^28,29^ This probably results from limited access to biological treatment in Poland (10% of patients from Poland used biological treatment in the present study).

Michael et al^30^ showed a lower annual cost of productivity loss due to absenteeism and presenteeism among Hungarian patients, as compared with our findings. This discrepancy is most likely due to differences in patient characteristics (eg, a lower proportion of patients with active disease, 26.9% vs 41.1% in our study) and in the method used for the valuation of productivity loss. This seems plausible because the mean absenteeism (CD, 8.5%; UC, 11.7%), presenteeism (CD, 27.6%; UC, 24.5%), and regular activity impairment (CD, 33.9%; UC, 31.0%) scores were similar between studies. In line with our findings, Michael et al reported that regular activity and work-related activity impairment were associated with disease severity (active disease or surgery in the past) and age (presenteeism and regular activity loss only) but not with IBD type.

Juan et al^5^ estimated the annual cost of absenteeism among Spanish patients with CD at €2218 (€3394 in 2019 €), which is lower than that in our study. However, the proportions of employed patients, patients with active disease or fistulas, and patients using biological treatment were significantly lower in the study by Juan et al.

## Conclusions

The results of our study indicate a high relevance of indirect cost (including informal care) in the context of economic analyses and can be used to inform the cost-effectiveness models of new treatment (eg, the costs can be implemented as a disease activity–dependent cost in a model) or to support the development of health policies, resource allocation, and patient care. Moreover, our findings suggest that future multinational studies should consider between-country variability during the assessment of work-related impairment or informal care because occupational and societal policies in a country can have significant impact on those aspects.

## Supplementary Material

izac144_suppl_Supplementary_Tables

## Data Availability

All data are presented in the manuscript or the Supplementary Appendix. The dataset is available from the corresponding author upon reasonable request.
