# Supplementary material for: Indirect Costs of Inflammatory Bowel Diseases: A Comparison of Patient-Reported Outcomes Across 12 European Countries
Source: Inflamm Bowel Dis. 2022 Jul 6;29(5):752–62. doi: 10.1093/ibd/izac144 (PMC10152296; doi:10.1093/ibd/izac144)
Supplement: izac144_suppl_Supplementary_Tables [file izac144_suppl_supplementary_tables.docx]

# Supplementary tables

## The outline of the questionnaires.

| **Language:** | **Questions / information in English** | **Choices in English** |
| --- | --- | --- |
| **Part 1.** | Lenguage selection page |  |
| **Part 2. General information about the study** | We invite you to participate in a questionnaire survey carried out by the Institute of Public Health, Jagiellonian University Collegium Medicum in cooperation with patient associations from European countries under the joint patronage of the *European Federation of Crohn's and ulcerative Colitis Associations* (EFCCA) |  |
|  | After reading the scope of the study and it’s aims, please give consent to participate in the study (select appropriate check box at the bottom of the page) or leave the page |  |
|  | The questionnaire is designed for **adults** with inflammatory bowel diseases (i.e. Crohn’s disease or ulcerative colitis) |  |
|  | Answers to questions included in the questionnaire are very important for the assessment of nonmedical costs of inflammatory bowel diseases in Europe. The aim of the study is to assess the influence of inflammatory bowel diseases on the patients productivity at work and relevant costs for the society. |  |
|  | Participation in the study is voluntary and anonymous. |  |
|  | No information identifying the participants is collected and no answer is required. |  |
|  |  | I agree to participate in the study and I state that I suffer from an inflammatory bowel disease |
| **Part 3. General information** | Please fill in the information below |  |
|  | Age in years |  |
|  | Sex | Male  Female |
|  | Country of residence | One of European countries |
|  | Place of residence | City of at least 100.000 habitants  City of no more than 100.000 habitants  Village |
|  | Which inflammatory bowel disease do you suffer from? | Crohn’s disease  Ulcerative colitis  Other inflammatory bowel disease |
|  | In every subsequent questions **„disease”** means inflammatory bowel disease indicated above. |  |
|  | Age at which the disease was diagnosed |  |
|  | Do you have any other chronic diseases? If so, please indicate them below. | I have no other chronic diseases.  I additionally suffer from (possible multiple choice ):  Certain infectious and parasitic diseases  Tumors (cancers)  Diseases of the blood and blood-forming organs and certain disorders involving the immune system  Endocrine, nutritional and metabolic diseases  Mental and behavioral disorders  Diseases of the nervous system  Diseases of the eye and adnexa  Diseases of the ear and mastoid process  Diseases of the circulatory system  Diseases of the respiratory system  Diseases of the digestive system  Diseases of the skin and subcutaneous tissue  Diseases of the musculoskeletal system and connective tissue  Diseases of the genitourinary system  Certain conditions originating in the perinatal period  Congenital malformations, deformations and chromosomal abnormalities |
| **Part 4** | Please indicate the number of medical consultations with a specialist due to the **disease** in the past month. |  |
|  | „Medical consultations” includes visits to a specialist’s office, a medical clinic, phone consultations, and home visits. Consultations with a primary health care specialist (family doctor), and phone conversations with a healthcare provider employee to set-up a consultation, and consultations during hospitalization for at least one day should not be taken into account. |  |
|  | Please indicate the number of days spent in a hospital due to the **disease** in the past month. |  |
|  | Please indicate all currently taken medication prescribed by a medical doctor. Possible multiple choice. | Sulfasalazine  Mesalazine  Prednisone, prednisolone or methylprednisolone  Budesonide  Azathioprine  6-mercaptopurine  Methotrexate  Adalimumab  Infliximab  Golimumab  Vedolizumab  Ciclosporin  Metronidazole  Beclomethasone dipropionate  Certolizumab  Ustekinumab  None of the above |
|  | When did you last undergo any surgical treatment due to the **disease**? | Never (I have never had any surgical treatment of an inflammatory bowel disease)  In the past year  In the past 5 years  Earlier |
| **Part 5** | During your last medical consultation, a medical doctor probably assessed your **disease**’s level of activity. Please indicate which answer best reflects the doctor’s opinion. |  |
|  | Remission of the disease (no symptoms) |  |
|  | Active disease (i.e. during a flare) |  |
| **P-HBI** | Please assess your general well-being during the past week on a scale from 1 (very bad) to 10 (very good). |  |
|  | Please assess the intensity of abdominal pain during the past week on scale from 1 (no pain) to 10 (the worst pain I can imagine). |  |
|  | Have you been diagnosed with a tumor/edema/intra-abdominal mass (palpable lump in the abdomen which can cause pain when pressed)? | Yes  No  I don’t know |
|  | On average per day, how many times have you had diarrhea (loose and watery stools) during the past week? |  |
|  | If you have a stoma or j-pouch please indicate only the higher frequency of stools or bag replacements in comparison to an ordinary week (0-no additional stools/bag replacements; e.g., if you indicate 3 it means that you have had 3 additional stools/bag replacements). |  |
|  | per day |  |
|  | Have you had the following symptoms? |  |
|  | Please indicate the best answer for each symptom. | Yes, presently / during the past week  Yes, earlier than a week ago  No, never  I don’t know |
|  | Joint pain, other than caused by sport activities, work or an accident |  |
|  | Eye infection diagnosed as uveitis by an eye-specialist |  |
|  | Skin disorder diagnosed as erythema nodosum |  |
|  | Skin disorder diagnosed as pyoderma |  |
|  | Mouth ulcers like aphthae (wounds or blisters in the mouth, also known as canker sores) |  |
|  | Rectal pain during bowel movements/anal fissure |  |
|  | Fistula with puss spill (active fistula) |  |
|  | Perianal abscess (painful ulcer in the rectal area) |  |
| **P-SCCAI** | On average per day, how many times did you use the toilet for defecation in the past week?  Blood and slime discharge is also considered defecation. | 0 to 3 times  From 4 to 6 times  From 7 to 9 times  More than 9 times |
|  | On average per night, how many times did you get out of bed to use the toilet for defecation in the past week? | Never  1 to 3 times  More than 3 times |
|  | During the past week, were you able to hold your stool for 15 minutes or longer, when you felt a strong urge to use the toilet? | Yes  No  I don’t know |
|  | During the past week, did you have to make adjustments to your activities, to ensure that there was a toilet nearby? | Yes  No  I don’t know |
|  | During the past week, have you found traces of stool on your underwear? | Yes  No  I don’t know |
|  | During the past week, how many times did you see blood in your stool? | Never  Much less often than in every other stool  A little less often than in every other stool  More often than in every other stool |
|  | If you had to rate your general well-being during the past week by giving it a number, what number would you choose? (1 = very bad, 10 = perfect) |  |
|  | Have you had the following symptoms? |  |
|  | Please indicate the best answer for each symptom. | Yes, presently / during past week  Yes, earlier than a week ago  No, never  I don’t know |
|  | Joint pain which was worse at rest than after an activity |  |
|  | Red or swollen joints |  |
|  | Woken up by joint pain |  |
|  | Skin disorder that has been diagnosed as erythema nodosum |  |
|  | Skin disorder that has been diagnosed as pyoderma |  |
|  | Eye infection diagnosed as uveïtis |  |
|  | Do you have a stoma (an opening in the abdominal wall with an external pouch) or j-pouch (an internal pouch formed from the small intestine)? | Yes  No |
| **Part 6** | On average per month, how much money do you spend on consultations with a specialist due to the **disease**? | 0 euro  More than 0 but less than 50 euro  More than 50 but less than 100 euro  More than 100 but less than 150 euro  More than 150 but less than 200 euro  More than 200 but less than 250 euro  More than 250 but less than 300 euro  More than 300 euro |
|  | On average per month, how much money do you spend on medication prescribed by a specialist due to the **disease**?  Please include only the cost of medication prescribed by a specialist due to the disease. If the amounts differ between months, please indicate the average value from the past 3 months. | 0 euro  More than 0 but less than 50 euro  More than 50 but less than 100 euro  More than 100 but less than 150 euro  More than 150 but less than 200 euro  More than 200 but less than 250 euro  More than 250 but less than 300 euro  More than 300 euro |
|  | On average per month, how much money do you spend on additional purchases due to the **disease**? Please, include all expenses which would not be incurred in case of lack of the disease. |  |
|  | a) expenses on: diet supplements and other formulations used in disease treatment, except medication prescribed by a specialist, dietary formulations due to a special diet, specialist equipment due to the disease, stoma bags, transport to specialist/hospital. | 0 euro  More than 0 but less than 50 euro  More than 50 but less than 100 euro  More than 100 but less than 150 euro  More than 150 but less than 200 euro  More than 200 but less than 250 euro  More than 250 but less than 300 euro  More than 300 euro |
|  | b) expenses on: materials about the disease (handbooks, scientific press, etc.) and additional hygienic articles due to the disease or other not listed above. | 0 euro  More than 0 but less than 50 euro  More than 50 but less than 100 euro  More than 100 but less than 150 euro  More than 150 but less than 200 euro  More than 200 but less than 250 euro  More than 250 but less than 300 euro  More than 300 euro |
| **Part 7** | Please, select the answer which best reflects your situation. Possible multiple choice. | I have paid work/I work for remuneration. Please also select if you take temporary jobs with remuneration, if you plan to continue such a form of work.  I am on short-term leave from work (maternity leave, sick leave, etc.)  Retired  Disability pension  Partly/completely unable to work due to the disease  Degree of disability  Currently studying  Registered as unemployed and looking for work  I do not work for other reasons (i.e. involuntary unemployment, volunteering, childcare, housework) |
| **Part 8** | “Work Productivity and Activity Impairment Questionnaire” (WPAI) | See: [Home (reillyassociates.net)](http://www.reillyassociates.net/WPAI_General.html) |
| **Part 9** | Please indicate the number of hours in a week that family members and/or non-relatives dedicate to assist you or care for you due to the **disease** without payment. |  |
|  | Please indicate the number of hours in a week that family members and/or non-relatives dedicate to assist you or care for you due to the **disease** without payment**,** leaving their own paid work. |  |

## Supplementary Table 1. Unit cost of productivity impairment.

|  | Unit cost (hour) of work lost (P4/P2/P5 x 0.65) | Unit cost (hour) of time lost outside work (P3) | P1. General population, thousands (2019)^1^ | P2. Worker population, thousands (2019)^1^ | P3. Compensation of employees per hour worked (2019)^3^ | P4. GDP, millions (2019)^2^ | P5. Working hours per year (2019)^1^ |
| --- | --- | --- | --- | --- | --- | --- | --- |
| Belgium | € 40.26 | € 39.90 | 11,489.00 | 4,895.20 | € 39.90 | € 478,160.70 | 1,576.9660 |
| Bulgaria | € 6.88 | € 6.10 | 6,975.76 | 3,533.58 | € 6.10 | € 61,558.00 | 1,645.2465 |
| Cyprus | € 18.57 | € 14.80 | 881.95 | 445.14 | € 14.80 | € 23,009.90 | 1,809.1477 |
| Czech Republic | € 15.12 | € 12.30 | 10,669.32 | 5,430.34 | € 12.30 | € 225,568.70 | 1,786.0348 |
| Denmark | € 49.00 | € 41.70 | 5,817.00 | 3,002.80 | € 41.70 | € 310,475.60 | 1,371.4993 |
| Greece | € 12.56 | € 10.20 | 10,721.55 | 4,691.10 | € 10.20 | € 183,250.40 | 2,021.9251 |
| Hungary | € 11.70 | € 8.30 | 9,771.14 | 4,715.24 | € 8.30 | € 146,113.20 | 1,722.2250 |
| Italy | € 26.75 | € 23.40 | 59,729.10 | 25,501.70 | € 23.40 | € 1,794,934.90 | 1,710.2118 |
| Poland | € 10.59 | € 8.10 | 38,386.00 | 16,397.90 | € 8.10 | € 533,599.90 | 1,996.8828 |
| Portugal | € 14.87 | € 12.10 | 10,286.30 | 4,952.80 | € 12.10 | € 214,374.60 | 1,891.8317 |
| Romania | € 9.30 | € 6.90 | 19,375.84 | 8,649.50 | € 6.90 | € 223,162.50 | 1,803.1122 |
| Spain | € 23.60 | € 20.50 | 47,105.36 | 20,364.60 | € 20.50 | € 1,244,375.00 | 1,682.9364 |

^1^ EUROSTAT database “Employment and population - selected international annual data” (naida_10_pe)

^2^ EUROSTAT database “GDP and main aggregates - selected international annual data” (naida_10_gdp)

^3^ EUROSTAT database “Labour productivity and unit labour costs” (nama_10_lp_ulc)

## Supplementary Table 2. Characteristics of all study participants by country.

|  | All patients | | | | | | | | | | | | | |
| --- | --- | --- | --- | --- | --- | --- | --- | --- | --- | --- | --- | --- | --- | --- |
|  | All | Belgium | Bulgaria | Cyprus | Czech Rep. | Denmark | Greece | Spain | Poland | Portugal | Romania | Hungary | Italy | P value |
| N | 3,687 | 128 | 141 | 53 | 69 | 1,253 | 264 | 257 | 467 | 651 | 131 | 77 | 196 | - |
| Age, mean (SD) | 43.03 (13.76) | 42.38 (13.75) | 41.18 (10.90) | 37.15 (12.74) | 37.90 (11.87) | 48.46 (14.61) | 39.78 (11.96) | 46.12 (11.62) | 37.90 (11.71) | 39.44 (12.04) | 38.20 (12.67) | 35.91 (10.24) | 43.78 (13.93) | <0.0001 |
| Male gender, n (%) | 1241 (34.0) | 43 (33.9) | 52 (37.7) | 24 (47.1) | 24 (34.8) | 369 (29.7) | 100 (38.5) | 99 (39.1) | 195 (42.0) | 183 (28.5) | 43 (32.8) | 26 (33.8) | 83 (42.4) | 0.0003 |
| Age at diagnosis, mean (SD) | 29.81 (12.42) | 27.68 (11.33) | 32.84 (11.33) | 26.88 (11.95) | 25.49 (10.55) | 32.26 (14.02) | 28.77 (11.93) | 29.15 (11.39) | 29.10 (11.14) | 27.51 (10.63) | 30.45 (12.95) | 25.97 (10.29) | 28.45 (11.29) | <0.0001 |
| *Place of residence, n (%)* |  |  |  |  |  |  |  |  |  |  |  |  |  |  |
| City with a population of ≥100 thousands | 1545 (42.2) | 39 (30.5) | 80 (58.0) | 27 (51.9) | 19 (27.9) | 405 (32.6) | 172 (65.2) | 91 (35.6) | 234 (50.1) | 304 (47.0) | 66 (51.2) | 48 (62.3) | 60 (30.9) | <0.0001 |
| City with a population of <100 thousands | 1242 (33.9) | 35 (27.3) | 50 (36.2) | 11 (21.2) | 25 (36.8) | 375 (30.2) | 63 (23.9) | 164 (64.1) | 156 (33.4) | 205 (31.7) | 45 (34.9) | 18 (23.4) | 95 (49.0) |  |
| Village | 876 (23.9) | 54 (42.2) | 8 (5.8) | 14 (26.9) | 24 (35.3) | 463 (37.3) | 29 (11.0) | 1 (0.4) | 77 (16.5) | 138 (21.3) | 18 (14.0) | 11 (14.3) | 39 (20.1) |  |
| *Disease, n (%)* |  |  |  |  |  |  |  |  |  |  |  |  |  |  |
| CD | 1930 (52.4) | 86 (67.2) | 54 (38.3) | 33 (62.3) | 41 (59.4) | 603 (48.1) | 179 (67.8) | 142 (55.3) | 186 (39.8) | 395 (60.7) | 61 (46.9) | 57 (74.0) | 93 (47.5) | <0.0001 |
| UC | 1693 (45.9) | 40 (31.3) | 84 (59.6) | 20 (37.7) | 27 (39.1) | 629 (50.2) | 84 (31.8) | 109 (42.4) | 273 (58.5) | 242 (37.2) | 66 (50.8) | 20 (26.0) | 99 (50.5) |  |
| Other IBD | 63 (1.7) | 2 (1.6) | 3 (2.1) | 0 (0.0) | 1 (1.5) | 21 (1.7) | 1 (0.4) | 6 (2.3) | 8 (1.7) | 14 (2.2) | 3 (2.3) | 0 (0.0) | 4 (2.0) |  |
| *Comorbidities, n (%)* |  |  |  |  |  |  |  |  |  |  |  |  |  |  |
| Any | 1893 (56.1) | 65 (55.1) | 80 (60.6) | 28 (58.3) | 43 (67.2) | 574 (51.0) | 144 (60.3) | 141 (59.0) | 287 (64.9) | 341 (57.2) | 57 (46.7) | 32 (46.4) | 101 (55.5) | 0.0071 |
| Diseases of the joints, musculoskeletal system or connective tissue | 527 (15.6) | 17 (14.4) | 11 (8.3) | 5 (10.4) | 15 (23.4) | 123 (10.9) | 50 (20.9) | 37 (15.5) | 84 (19.0) | 133 (22.3) | 5 (4.1) | 6 (8.7) | 41 (22.5) | <0.0001 |
| Diseases of the eye | 256 (7.6) | 7 (5.9) | 7 (5.3) | 4 (8.3) | 8 (12.5) | 43 (3.8) | 26 (10.9) | 19 (8.0) | 54 (12.2) | 60 (10.1) | 13 (10.7) | 2 (2.9) | 13 (7.1) | 0.0001 |
| Liver or bile duct diseases | 217 (6.4) | 2 (1.7) | 17 (12.9) | 1 (2.1) | 6 (9.4) | 54 (4.8) | 14 (5.9) | 14 (5.9) | 49 (11.1) | 33 (5.5) | 13 (10.7) | 4 (5.8) | 10 (5.5) | 0.0035 |
| Other than IBD diseases of the digestive system | 242 (7.2) | 13 (11.0) | 14 (10.6) | 4 (8.3) | 5 (7.8) | 56 (5.0) | 26 (10.9) | 23 (9.6) | 37 (8.4) | 35 (5.9) | 6 (4.9) | 7 (10.1) | 16 (8.8) | >0.9999 |
| Diseases of the skin and subcutaneous tissue | 472 (14.0) | 13 (11.0) | 16 (12.1) | 1 (2.1) | 15 (23.4) | 167 (14.8) | 27 (11.3) | 38 (15.9) | 67 (15.2) | 87 (14.6) | 19 (15.6) | 2 (2.9) | 20 (11.0) | >0.9999 |
| Diseases of the bones - osteoporosis | 356 (10.6) | 13 (11.0) | 18 (13.6) | 8 (16.7) | 12 (18.8) | 80 (7.1) | 42 (17.6) | 36 (15.1) | 42 (9.5) | 55 (9.2) | 22 (18.0) | 4 (5.8) | 24 (13.2) | 0.0001 |
| Kidney diseases | 145 (4.3) | 5 (4.2) | 10 (7.6) | 2 (4.2) | 4 (6.3) | 33 (2.9) | 5 (2.1) | 15 (6.3) | 20 (4.5) | 32 (5.4) | 9 (7.4) | 0 (0.0) | 10 (5.5) | >0.9999 |
| Tumor (cancer) | 63 (1.9) | 3 (2.5) | 2 (1.5) | 0 (0.0) | 1 (1.6) | 20 (1.8) | 3 (1.3) | 3 (1.3) | 11 (2.5) | 13 (2.2) | 2 (1.6) | 1 (1.5) | 4 (2.2) | >0.9999 |
| Diseases of the blood or blood-forming organs or certain disorders involving the immune system | 148 (4.4) | 8 (6.8) | 13 (9.9) | 1 (2.1) | 5 (7.8) | 26 (2.3) | 9 (3.8) | 10 (4.2) | 38 (8.6) | 23 (3.9) | 6 (4.9) | 1 (1.5) | 8 (4.4) | 0.0007 |
| Endocrine, nutritional or metabolic diseases | 324 (9.6) | 9 (7.6) | 17 (12.9) | 6 (12.5) | 7 (10.9) | 66 (5.9) | 38 (15.9) | 23 (9.6) | 72 (16.3) | 46 (7.7) | 16 (13.1) | 10 (14.5) | 14 (7.7) | <0.0001 |
| Mental and behavioral disorders | 237 (7.0) | 4 (3.4) | 7 (5.3) | 7 (14.6) | 8 (12.5) | 87 (7.7) | 40 (16.7) | 25 (10.5) | 19 (4.3) | 26 (4.4) | 3 (2.5) | 6 (8.7) | 5 (2.8) | <0.0001 |
| Diseases of the nervous system | 168 (5.0) | 3 (2.5) | 10 (7.6) | 6 (12.5) | 5 (7.8) | 34 (3.0) | 12 (5.0) | 9 (3.8) | 26 (5.9) | 47 (7.9) | 5 (4.1) | 5 (7.3) | 6 (3.3) | 0.0873 |
| Diseases of the ear | 76 (2.3) | 1 (0.9) | 3 (2.3) | 0 (0.0) | 3 (4.7) | 25 (2.2) | 7 (2.9) | 6 (2.5) | 7 (1.6) | 17 (2.9) | 0 (0.0) | 1 (1.5) | 6 (3.3) | >0.9999 |
| Diseases of the circulatory system | 184 (5.5) | 1 (0.9) | 6 (4.6) | 2 (4.2) | 2 (3.1) | 54 (4.8) | 7 (2.9) | 15 (6.3) | 48 (10.9) | 24 (4.0) | 6 (4.9) | 5 (7.3) | 14 (7.7) | 0.0078 |
| Diseases of the respiratory system | 297 (8.8) | 10 (8.5) | 9 (6.8) | 2 (4.2) | 7 (10.9) | 112 (10.0) | 15 (6.3) | 22 (9.2) | 35 (7.9) | 58 (9.7) | 14 (11.5) | 1 (1.5) | 12 (6.6) | >0.9999 |
| Diseases of the genitourinary system | 118 (3.5) | 2 (1.7) | 7 (5.3) | 2 (4.2) | 7 (10.9) | 12 (1.1) | 8 (3.4) | 9 (3.8) | 29 (6.6) | 19 (3.2) | 7 (5.7) | 3 (4.4) | 13 (7.1) | 0.0001 |
| Congenital malformations, deformations and/or chromosomal abnormalities | 18 (0.5) | 2 (1.7) | 1 (0.8) | 0 (0.0) | 1 (1.6) | 6 (0.5) | 0 (0.0) | 0 (0.0) | 3 (0.7) | 2 (0.3) | 2 (1.6) | 1 (1.5) | 0 (0.0) | >0.9999 |
| Infectious or parasitic diseases | 64 (1.9) | 4 (3.4) | 4 (3.0) | 4 (8.3) | 1 (1.6) | 10 (0.9) | 3 (1.3) | 5 (2.1) | 22 (5.0) | 6 (1.0) | 3 (2.5) | 1 (1.5) | 1 (0.6) | 0.0005 |
| *Current pharmacotherapy, n (%)* |  |  |  |  |  |  |  |  |  |  |  |  |  |  |
| Any | 3074 (84.9) | 112 (88.9) | 112 (79.4) | 43 (82.7) | 54 (79.4) | 938 (77.0) | 236 (91.8) | 225 (87.6) | 418 (90.5) | 580 (90.3) | 127 (98.5) | 55 (74.3) | 174 (89.7) | <0.0001 |
| Sulfasalazine | 416 (11.5) | 12 (9.5) | 46 (32.6) | 5 (9.6) | 6 (8.8) | 94 (7.7) | 11 (4.3) | 10 (3.9) | 98 (21.2) | 84 (13.1) | 30 (23.3) | 11 (14.9) | 9 (4.6) | <0.0001 |
| Mesalazine | 1537 (42.5) | 45 (35.7) | 56 (39.7) | 22 (42.3) | 26 (38.2) | 410 (33.7) | 80 (31.1) | 91 (35.4) | 297 (64.3) | 279 (43.5) | 80 (62.0) | 30 (40.5) | 121 (62.4) | <0.0001 |
| Plain steroids | 653 (18.0) | 16 (12.7) | 34 (24.1) | 6 (11.5) | 19 (27.9) | 382 (31.4) | 13 (5.1) | 14 (5.5) | 28 (6.1) | 51 (7.9) | 63 (48.8) | 7 (9.5) | 20 (10.3) | <0.0001 |
| Budesonide | 255 (7.0) | 11 (8.7) | 10 (7.1) | 1 (1.9) | 9 (13.2) | 81 (6.7) | 12 (4.7) | 6 (2.3) | 36 (7.8) | 40 (6.2) | 27 (20.9) | 15 (20.3) | 7 (3.6) | <0.0001 |
| Azathioprine | 966 (26.7) | 30 (23.8) | 33 (23.4) | 15 (28.9) | 14 (20.6) | 298 (24.5) | 82 (31.9) | 71 (27.6) | 144 (31.2) | 203 (31.6) | 34 (26.4) | 20 (27.0) | 22 (11.3) | 0.0008 |
| Mercaptopurine | 96 (2.7) | 7 (5.6) | 0 (0.0) | 1 (1.9) | 0 (0.0) | 46 (3.8) | 1 (0.4) | 7 (2.7) | 27 (5.8) | 3 (0.5) | 3 (2.3) | 0 (0.0) | 1 (0.5) | <0.0001 |
| Methotrexate | 143 (4.0) | 4 (3.2) | 1 (0.7) | 1 (1.9) | 1 (1.5) | 75 (6.2) | 17 (6.6) | 12 (4.7) | 8 (1.7) | 16 (2.5) | 5 (3.9) | 0 (0.0) | 3 (1.6) | 0.0031 |
| Adalimumab | 357 (9.9) | 22 (17.5) | 18 (12.8) | 0 (0.0) | 6 (8.8) | 53 (4.4) | 26 (10.1) | 57 (22.2) | 7 (1.5) | 93 (14.5) | 43 (33.3) | 9 (12.2) | 23 (11.9) | <0.0001 |
| Infliximab | 569 (15.7) | 21 (16.7) | 2 (1.4) | 9 (17.3) | 9 (13.2) | 221 (18.1) | 49 (19.1) | 37 (14.4) | 27 (5.8) | 132 (20.6) | 37 (28.7) | 4 (5.4) | 21 (10.8) | <0.0001 |
| Golimumab | 24 (0.7) | 1 (0.8) | 1 (0.7) | 0 (0.0) | 0 (0.0) | 7 (0.6) | 5 (2.0) | 5 (2.0) | 0 (0.0) | 3 (0.5) | 1 (0.8) | 0 (0.0) | 1 (0.5) | >0.9999 |
| Vedolizumab | 151 (4.2) | 13 (10.3) | 2 (1.4) | 1 (1.9) | 3 (4.4) | 30 (2.5) | 13 (5.1) | 20 (7.8) | 8 (1.7) | 39 (6.1) | 1 (0.8) | 5 (6.8) | 16 (8.3) | <0.0001 |
| Ciclosporin | 21 (0.6) | 0 (0.0) | 3 (2.1) | 0 (0.0) | 0 (0.0) | 5 (0.4) | 0 (0.0) | 2 (0.8) | 1 (0.2) | 4 (0.6) | 3 (2.3) | 0 (0.0) | 3 (1.6) | >0.9999 |
| Metronidazole | 147 (4.1) | 2 (1.6) | 8 (5.7) | 2 (3.9) | 1 (1.5) | 55 (4.5) | 3 (1.2) | 4 (1.6) | 21 (4.6) | 15 (2.3) | 30 (23.3) | 1 (1.4) | 5 (2.6) | <0.0001 |
| Beclomethasone | 19 (0.5) | 2 (1.6) | 0 (0.0) | 0 (0.0) | 0 (0.0) | 4 (0.3) | 0 (0.0) | 1 (0.4) | 1 (0.2) | 1 (0.2) | 0 (0.0) | 0 (0.0) | 10 (5.2) | <0.0001 |
| Certolizumab | 5 (0.1) | 0 (0.0) | 0 (0.0) | 0 (0.0) | 0 (0.0) | 2 (0.2) | 1 (0.4) | 0 (0.0) | 1 (0.2) | 0 (0.0) | 1 (0.8) | 0 (0.0) | 0 (0.0) | >0.9999 |
| Ustekinumab | 77 (2.1) | 15 (11.9) | 0 (0.0) | 1 (1.9) | 4 (5.9) | 14 (1.2) | 8 (3.1) | 17 (6.6) | 1 (0.2) | 5 (0.8) | 0 (0.0) | 1 (1.4) | 11 (5.7) | <0.0001 |
| Any biological treatment | 1123 (31.0) | 72 (57.1) | 23 (16.3) | 11 (21.2) | 21 (30.9) | 296 (24.3) | 102 (39.7) | 132 (51.4) | 44 (9.5) | 266 (41.4) | 66 (51.2) | 18 (24.3) | 72 (37.1) | <0.0001 |
| *Past surgical treatment, n (%)* |  |  |  |  |  |  |  |  |  |  |  |  |  |  |
| previous year | 268 (7.3) | 13 (10.2) | 6 (4.3) | 1 (2.0) | 8 (11.6) | 87 (7.0) | 22 (8.5) | 13 (5.1) | 35 (7.5) | 43 (6.6) | 13 (10.0) | 8 (10.5) | 19 (9.7) | <0.0001 |
| 1 to 5 years ago | 461 (12.6) | 20 (15.6) | 10 (7.1) | 1 (2.0) | 14 (20.3) | 156 (12.5) | 33 (12.7) | 43 (17.0) | 63 (13.6) | 64 (9.9) | 22 (16.9) | 12 (15.8) | 23 (11.8) |  |
| 5+ years ago | 580 (15.8) | 22 (17.2) | 15 (10.6) | 2 (4.0) | 10 (14.5) | 246 (19.7) | 23 (8.9) | 53 (21.0) | 33 (7.1) | 120 (18.5) | 4 (3.1) | 16 (21.1) | 36 (18.5) |  |
| *Disease activity at last clinical assessment, n (%)* |  |  |  |  |  |  |  |  |  |  |  |  |  |  |
| Remission | 1989 (54.3) | 64 (50.0) | 55 (39.3) | 28 (54.9) | 39 (56.5) | 772 (62.0) | 170 (64.9) | 162 (63.3) | 250 (54.2) | 256 (39.4) | 60 (47.6) | 48 (63.2) | 85 (43.4) | <0.0001 |
| Active disease | 1490 (40.7) | 60 (46.9) | 73 (52.1) | 21 (41.2) | 29 (42.0) | 399 (32.0) | 86 (32.8) | 83 (32.4) | 188 (40.8) | 360 (55.4) | 59 (46.8) | 27 (35.5) | 105 (53.6) |  |
| 'don't know'/ ‘don’t remember’ | 182 (5.0) | 4 (3.1) | 12 (8.6) | 2 (3.9) | 1 (1.5) | 75 (6.0) | 6 (2.3) | 11 (4.3) | 23 (5.0) | 34 (5.2) | 7 (5.6) | 1 (1.3) | 6 (3.1) |  |
| *Time of the last assessment of disease activity, n (%)* |  |  |  |  |  |  |  |  |  |  |  |  |  |  |
| previous month | 1307 (35.9) | 53 (41.4) | 39 (28.3) | 14 (28.6) | 32 (46.4) | 316 (25.5) | 122 (46.9) | 85 (33.7) | 201 (43.5) | 289 (44.5) | 50 (40.0) | 27 (35.5) | 79 (40.7) | <0.0001 |
| 1-2 months ago | 637 (17.5) | 24 (18.8) | 17 (12.3) | 11 (22.5) | 12 (17.4) | 224 (18.1) | 34 (13.1) | 53 (21.0) | 84 (18.2) | 104 (16.0) | 18 (14.4) | 13 (17.1) | 43 (22.2) |  |
| 2-3 months ago | 581 (16.0) | 22 (17.2) | 22 (15.9) | 11 (22.5) | 12 (17.4) | 182 (14.7) | 41 (15.8) | 75 (29.8) | 63 (13.6) | 99 (15.2) | 22 (17.6) | 7 (9.2) | 25 (12.9) |  |
| 3+ months ago | 1118 (30.7) | 29 (22.7) | 60 (43.5) | 13 (26.5) | 13 (18.8) | 518 (41.8) | 63 (24.2) | 39 (15.5) | 114 (24.7) | 158 (24.3) | 35 (28.0) | 29 (38.2) | 47 (24.2) |  |
| *Current disease activity, n (%)* |  |  |  |  |  |  |  |  |  |  |  |  |  |  |
| Remission | 2160 (58.9) | 52 (40.6) | 91 (64.5) | 20 (39.2) | 23 (33.8) | 825 (66.1) | 138 (52.9) | 143 (55.6) | 282 (60.5) | 362 (55.8) | 72 (56.3) | 43 (56.6) | 109 (55.6) | <0.0001 |
| Active disease | 1509 (41.1) | 76 (59.4) | 50 (35.5) | 31 (60.8) | 45 (66.2) | 423 (33.9) | 123 (47.1) | 114 (44.4) | 184 (39.5) | 287 (44.2) | 56 (43.8) | 33 (43.4) | 87 (44.4) |  |
| *Current activity of CD or other IBD, n (%)* |  |  |  |  |  |  |  |  |  |  |  |  |  |  |
| Remission | 923 (46.5) | 28 (31.8) | 29 (50.9) | 11 (34.4) | 9 (21.4) | 331 (53.2) | 85 (47.5) | 61 (41.2) | 84 (43.3) | 188 (46.2) | 32 (50.8) | 29 (51.8) | 36 (37.1) | 0.0109 |
| Active disease | 1062 (53.5) | 60 (68.2) | 28 (49.1) | 21 (65.6) | 33 (78.6) | 291 (46.8) | 94 (52.5) | 87 (58.8) | 110 (56.7) | 219 (53.8) | 31 (49.2) | 27 (48.2) | 61 (62.9) |  |
| P-HBI score, mean (SD) | 6.00 (5.45) | 7.56 (5.25) | 5.28 (4.17) | 6.38 (4.37) | 7.79 (5.11) | 5.56 (5.47) | 5.53 (4.28) | 7.54 (8.25) | 6.22 (5.14) | 5.63 (4.88) | 6.02 (6.20) | 5.43 (5.59) | 6.85 (4.76) | 0.0022 |
| *Current activity of UC, n (%)* |  |  |  |  |  |  |  |  |  |  |  |  |  |  |
| Remission | 1237 (73.5) | 24 (60.0) | 62 (73.8) | 9 (47.4) | 14 (53.9) | 494 (78.9) | 53 (64.6) | 82 (75.2) | 198 (72.8) | 174 (71.9) | 40 (61.5) | 14 (70.0) | 73 (73.7) | 0.0806 |
| Active disease | 447 (26.5) | 16 (40.0) | 22 (26.2) | 10 (52.6) | 12 (46.2) | 132 (21.1) | 29 (35.4) | 27 (24.8) | 74 (27.2) | 68 (28.1) | 25 (38.5) | 6 (30.0) | 26 (26.3) |  |
| P-SCCAI score (UC), mean (SD) | 3.78 (3.24) | 4.80 (4.01) | 3.62 (3.55) | 5.68 (3.71) | 5.42 (4.29) | 3.33 (2.93) | 4.23 (3.20) | 3.74 (2.98) | 4.05 (3.44) | 3.79 (3.06) | 3.95 (3.40) | 4.30 (4.54) | 4.26 (3.45) | 0.8370 |
| UC patients with stoma, n (%) | 132 (7.8) | 1 (2.5) | 0 (0.0) | 2 (10.0) | 1 (3.7) | 54 (8.6) | 10 (12.1) | 17 (15.6) | 11 (4.0) | 13 (5.4) | 10 (15.2) | 4 (20.0) | 9 (9.1) | 0.0163 |
| Penetrating CD course, n (%) | 595 (30.9) | 25 (29.4) | 15 (28.3) | 5 (17.9) | 20 (48.8) | 207 (33.7) | 43 (25.9) | 25 (18.0) | 55 (29.0) | 129 (32.2) | 15 (25.0) | 20 (37.0) | 36 (39.1) | 0.4391 |
| Employed, n (%) | 2455 (67.0) | 73 (57.0) | 104 (73.8) | 32 (64.0) | 48 (69.6) | 833 (66.9) | 127 (48.9) | 171 (66.5) | 349 (74.9) | 472 (73.0) | 74 (57.8) | 57 (75.0) | 115 (59.0) | <0.0001 |
| During short-term absence from work, n (%) | 407 (11.0) | 13 (10.2) | 28 (19.9) | 5 (9.4) | 9 (13.0) | 116 (9.3) | 15 (5.7) | 27 (10.5) | 49 (10.5) | 97 (14.9) | 13 (9.9) | 10 (13.0) | 25 (12.8) | 0.0576 |
| Retired, n (%) | 380 (10.3) | 15 (11.7) | 7 (5.0) | 2 (3.8) | 2 (2.9) | 203 (16.2) | 14 (5.3) | 23 (9.0) | 28 (6.0) | 38 (5.8) | 25 (19.1) | 2 (2.6) | 21 (10.7) | <0.0001 |
| On a disability pension, n (%) | 384 (10.4) | 6 (4.7) | 29 (20.6) | 2 (3.8) | 25 (36.2) | 175 (14.0) | 42 (15.9) | 24 (9.3) | 33 (7.1) | 14 (2.2) | 9 (6.9) | 14 (18.2) | 11 (5.6) | <0.0001 |
| Unable to work due to IBD, n (%) | 343 (9.3) | 36 (28.1) | 16 (11.4) | 4 (7.6) | 8 (11.6) | 88 (7.0) | 23 (8.7) | 26 (10.1) | 56 (12.0) | 32 (4.9) | 16 (12.2) | 10 (13.0) | 28 (14.3) | <0.0001 |
| With disability certificate | 523 (14.2) | 13 (10.2) | 9 (6.4) | 2 (3.8) | 7 (10.1) | 18 (1.4) | 58 (22.0) | 90 (35.0) | 161 (34.5) | 82 (12.6) | 8 (6.1) | 4 (5.2) | 71 (36.2) | <0.0001 |
| Student, n (%) | 312 (8.5) | 5 (3.9) | 9 (6.4) | 5 (9.4) | 8 (11.6) | 87 (6.9) | 27 (10.2) | 19 (7.4) | 40 (8.6) | 65 (10.0) | 18 (13.7) | 7 (9.1) | 22 (11.2) | >0.9999 |
| Registered unemployment, n (%) | 180 (4.9) | 2 (1.6) | 5 (3.6) | 3 (5.7) | 3 (4.4) | 32 (2.6) | 37 (14.0) | 19 (7.4) | 11 (2.4) | 47 (7.2) | 0 (0.0) | 2 (2.6) | 19 (9.7) | <0.0001 |
| Not registered unemployment, n (%) | 160 (4.3) | 1 (0.8) | 13 (9.2) | 2 (3.8) | 1 (1.5) | 32 (2.6) | 19 (7.2) | 6 (2.3) | 26 (5.6) | 35 (5.4) | 9 (6.9) | 3 (3.9) | 13 (6.6) | 0.0122 |
| Number of consultations with clinician in previous month, mean (SD) | 1.84 (3.47) | 2.25 (2.42) | 2.25 (4.35) | 1.69 (2.19) | 1.83 (2.29) | 1.23 (3.11) | 1.98 (2.72) | 5.43 (5.46) | 1.54 (2.88) | 1.56 (3.07) | 2.45 (4.35) | 1.79 (2.52) | 1.63 (3.04) | <0.0001 |
| Length of hospital stay in previous month, mean (SD) | 0.89 (2.85) | 1.12 (2.41) | 1.38 (3.75) | 1.29 (4.08) | 0.52 (1.98) | 0.72 (2.11) | 0.61 (2.43) | 0.67 (3.58) | 0.78 (2.97) | 1.23 (3.42) | 1.90 (3.96) | 0.30 (1.07) | 0.78 (2.81) | <0.0001 |

## Supplementary Table 3. Characteristics of employed study participants by country.

|  | Employed patients | | | | | | | | | | | | | |
| --- | --- | --- | --- | --- | --- | --- | --- | --- | --- | --- | --- | --- | --- | --- |
|  | All | Belgium | Bulgaria | Cyprus | Czech Rep. | Denmark | Greece | Spain | Poland | Portugal | Romania | Hungary | Italy | P value |
| N | 2,455 | 73 | 104 | 32 | 48 | 833 | 127 | 171 | 349 | 472 | 74 | 57 | 115 | - |
| Age, mean (SD) | 41.40 (10.91) | 39.48 (10.19) | 40.93 (9.52) | 36.94 (9.43) | 37.56 (10.13) | 45.16 (11.69) | 38.74 (8.50) | 45.01 (9.22) | 37.19 (9.66) | 39.45 (9.92) | 36.96 (8.82) | 35.74 (9.09) | 42.70 (10.71) | <0.0001 |
| Male gender, n (%) | 848 (34.9) | 23 (31.5) | 38 (37.6) | 14 (45.2) | 18 (37.5) | 241 (29.1) | 62 (49.2) | 63 (37.5) | 154 (44.5) | 138 (29.7) | 27 (36.5) | 17 (29.8) | 53 (46.1) | 0.0001 |
| Age at diagnosis, mean (SD) | 28.94 (10.61) | 28.12 (10.31) | 32.80 (9.86) | 25.78 (8.72) | 26.25 (10.44) | 30.17 (11.86) | 27.28 (8.70) | 28.30 (10.58) | 28.72 (9.59) | 27.62 (9.67) | 29.55 (8.84) | 27.14 (10.29) | 28.41 (10.60) | <0.0001 |
| *Place of residence, n (%)* |  |  |  |  |  |  |  |  |  |  |  |  |  |  |
| City with a population of ≥100 thousands | 1033 (42.3) | 25 (34.3) | 61 (59.8) | 17 (54.8) | 13 (27.7) | 275 (33.2) | 86 (67.7) | 56 (32.9) | 177 (50.7) | 218 (46.6) | 37 (50.7) | 35 (61.4) | 33 (29.0) | <0.0001 |
| City with a population of <100 thousands | 848 (34.8) | 21 (28.8) | 34 (33.3) | 8 (25.8) | 21 (44.7) | 254 (30.6) | 33 (26.0) | 114 (67.1) | 119 (34.1) | 141 (30.1) | 27 (37.0) | 12 (21.1) | 64 (56.1) |  |
| Village | 559 (22.9) | 27 (37.0) | 7 (6.9) | 6 (19.4) | 13 (27.7) | 300 (36.2) | 8 (6.3) | 0 (0.0) | 53 (15.2) | 109 (23.3) | 9 (12.3) | 10 (17.5) | 17 (14.9) |  |
| *Disease, n (%)* |  |  |  |  |  |  |  |  |  |  |  |  |  |  |
| CD | 1230 (50.1) | 45 (61.6) | 36 (34.6) | 18 (56.3) | 26 (54.2) | 390 (46.8) | 76 (59.8) | 92 (53.8) | 126 (36.1) | 291 (61.7) | 31 (41.9) | 43 (75.4) | 56 (48.7) | <0.0001 |
| UC | 1194 (48.6) | 27 (37.0) | 66 (63.5) | 14 (43.8) | 21 (43.8) | 434 (52.1) | 51 (40.2) | 77 (45.0) | 218 (62.5) | 174 (36.9) | 41 (55.4) | 14 (24.6) | 57 (49.6) |  |
| Other IBD | 31 (1.3) | 1 (1.4) | 2 (1.9) | 0 (0.0) | 1 (2.1) | 9 (1.1) | 0 (0.0) | 2 (1.2) | 5 (1.4) | 7 (1.5) | 2 (2.7) | 0 (0.0) | 2 (1.7) |  |
| *Comorbidities, n (%)* |  |  |  |  |  |  |  |  |  |  |  |  |  |  |
| Any | 1173 (52.9) | 29 (44.6) | 56 (57.7) | 14 (48.3) | 29 (65.9) | 341 (46.3) | 69 (61.6) | 85 (54.5) | 206 (63.2) | 236 (55.4) | 24 (35.8) | 25 (49.0) | 59 (55.1) | 0.0008 |
| Diseases of the joints, musculoskeletal system or connective tissue | 316 (14.3) | 7 (10.8) | 6 (6.2) | 4 (13.8) | 8 (18.2) | 68 (9.2) | 22 (19.6) | 23 (14.7) | 54 (16.6) | 91 (21.4) | 3 (4.5) | 3 (5.9) | 27 (25.2) | <0.0001 |
| Diseases of the eye | 155 (7.0) | 3 (4.6) | 5 (5.2) | 1 (3.5) | 4 (9.1) | 24 (3.3) | 10 (8.9) | 13 (8.3) | 35 (10.7) | 45 (10.6) | 7 (10.5) | 1 (2.0) | 7 (6.5) | 0.0200 |
| Liver or bile duct diseases | 132 (6.0) | 0 (0.0) | 12 (12.4) | 1 (3.5) | 5 (11.4) | 34 (4.6) | 4 (3.6) | 8 (5.1) | 35 (10.7) | 20 (4.7) | 3 (4.5) | 3 (5.9) | 7 (6.5) | 0.1328 |
| Other than IBD diseases of the digestive system | 149 (6.7) | 5 (7.7) | 11 (11.3) | 3 (10.3) | 3 (6.8) | 30 (4.1) | 11 (9.8) | 13 (8.3) | 29 (8.9) | 26 (6.1) | 2 (3.0) | 7 (13.7) | 9 (8.4) | >0.9999 |
| Diseases of the skin and subcutaneous tissue | 297 (13.4) | 7 (10.8) | 11 (11.3) | 0 (0.0) | 10 (22.7) | 101 (13.7) | 13 (11.6) | 22 (14.1) | 48 (14.7) | 66 (15.5) | 9 (13.4) | 0 (0.0) | 10 (9.4) | >0.9999 |
| Diseases of the bones - osteoporosis | 183 (8.3) | 7 (10.8) | 11 (11.3) | 3 (10.3) | 9 (20.5) | 43 (5.8) | 17 (15.2) | 19 (12.2) | 23 (7.1) | 34 (8.0) | 4 (6.0) | 3 (5.9) | 10 (9.4) | 0.6131 |
| Kidney diseases | 76 (3.4) | 2 (3.1) | 5 (5.2) | 1 (3.5) | 3 (6.8) | 14 (1.9) | 2 (1.8) | 8 (5.1) | 12 (3.7) | 22 (5.2) | 2 (3.0) | 0 (0.0) | 5 (4.7) | >0.9999 |
| Tumor (cancer) | 28 (1.3) | 0 (0.0) | 1 (1.0) | 0 (0.0) | 0 (0.0) | 5 (0.7) | 1 (0.9) | 0 (0.0) | 7 (2.2) | 8 (1.9) | 1 (1.5) | 1 (2.0) | 4 (3.7) | >0.9999 |
| Diseases of the blood or blood-forming organs or certain disorders involving the immune system | 72 (3.3) | 2 (3.1) | 6 (6.2) | 0 (0.0) | 2 (4.6) | 11 (1.5) | 5 (4.5) | 8 (5.1) | 20 (6.1) | 11 (2.6) | 2 (3.0) | 1 (2.0) | 4 (3.7) | >0.9999 |
| Endocrine, nutritional or metabolic diseases | 203 (9.2) | 3 (4.6) | 14 (14.4) | 2 (6.9) | 4 (9.1) | 41 (5.6) | 19 (17.0) | 12 (7.7) | 47 (14.4) | 35 (8.2) | 7 (10.5) | 8 (15.7) | 11 (10.3) | 0.0098 |
| Mental and behavioral disorders | 122 (5.5) | 1 (1.5) | 4 (4.1) | 5 (17.2) | 6 (13.6) | 40 (5.4) | 20 (17.9) | 13 (8.3) | 11 (3.4) | 13 (3.1) | 1 (1.5) | 5 (9.8) | 3 (2.8) | <0.0001 |
| Diseases of the nervous system | 80 (3.6) | 1 (1.5) | 5 (5.2) | 4 (13.8) | 1 (2.3) | 14 (1.9) | 5 (4.5) | 5 (3.2) | 14 (4.3) | 23 (5.4) | 2 (3.0) | 4 (7.8) | 2 (1.9) | >0.9999 |
| Diseases of the ear | 41 (1.9) | 0 (0.0) | 3 (3.1) | 0 (0.0) | 2 (4.6) | 11 (1.5) | 3 (2.7) | 6 (3.9) | 1 (0.3) | 13 (3.1) | 0 (0.0) | 1 (2.0) | 1 (0.9) | >0.9999 |
| Diseases of the circulatory system | 95 (4.3) | 0 (0.0) | 4 (4.1) | 1 (3.5) | 1 (2.3) | 19 (2.6) | 3 (2.7) | 9 (5.8) | 31 (9.5) | 16 (3.8) | 2 (3.0) | 2 (3.9) | 7 (6.5) | 0.0798 |
| Diseases of the respiratory system | 172 (7.8) | 7 (10.8) | 5 (5.2) | 1 (3.5) | 4 (9.1) | 65 (8.8) | 4 (3.6) | 15 (9.6) | 23 (7.1) | 37 (8.7) | 3 (4.5) | 1 (2.0) | 7 (6.5) | >0.9999 |
| Diseases of the genitourinary system | 65 (2.9) | 1 (1.5) | 4 (4.1) | 0 (0.0) | 2 (4.6) | 9 (1.2) | 5 (4.5) | 5 (3.2) | 17 (5.2) | 12 (2.8) | 2 (3.0) | 3 (5.9) | 5 (4.7) | >0.9999 |
| Congenital malformations, deformations and/or chromosomal abnormalities | 13 (0.6) | 2 (3.1) | 1 (1.0) | 0 (0.0) | 0 (0.0) | 4 (0.5) | 0 (0.0) | 0 (0.0) | 2 (0.6) | 1 (0.2) | 2 (3.0) | 1 (2.0) | 0 (0.0) | >0.9999 |
| Infectious or parasitic diseases | 44 (2.0) | 2 (3.1) | 3 (3.1) | 1 (3.5) | 1 (2.3) | 8 (1.1) | 2 (1.8) | 3 (1.9) | 17 (5.2) | 4 (0.9) | 1 (1.5) | 1 (2.0) | 1 (0.9) | >0.9999 |
| *Current pharmacotherapy, n (%)* |  |  |  |  |  |  |  |  |  |  |  |  |  |  |
| Any | 2073 (85.7) | 71 (97.3) | 85 (81.7) | 27 (84.4) | 38 (79.2) | 636 (78.5) | 115 (92.0) | 146 (85.4) | 318 (91.9) | 425 (90.8) | 72 (97.3) | 40 (72.7) | 100 (87.7) | <0.0001 |
| Sulfasalazine | 291 (12.0) | 6 (8.2) | 36 (34.6) | 3 (9.4) | 4 (8.3) | 61 (7.5) | 7 (5.6) | 6 (3.5) | 75 (21.7) | 60 (12.8) | 21 (28.4) | 6 (10.9) | 6 (5.3) | <0.0001 |
| Mesalazine | 1067 (44.1) | 26 (35.6) | 46 (44.2) | 15 (46.9) | 17 (35.4) | 289 (35.7) | 48 (38.4) | 58 (33.9) | 231 (66.8) | 195 (41.7) | 49 (66.2) | 23 (41.8) | 70 (61.4) | <0.0001 |
| Plain steroids | 403 (16.7) | 8 (11.0) | 24 (23.1) | 4 (12.5) | 14 (29.2) | 243 (30.0) | 5 (4.0) | 9 (5.3) | 16 (4.6) | 35 (7.5) | 31 (41.9) | 5 (9.1) | 9 (7.9) | <0.0001 |
| Budesonide | 169 (7.0) | 4 (5.5) | 9 (8.7) | 0 (0.0) | 5 (10.4) | 51 (6.3) | 7 (5.6) | 5 (2.9) | 28 (8.1) | 25 (5.3) | 17 (23.0) | 13 (23.6) | 5 (4.4) | <0.0001 |
| Azathioprine | 658 (27.2) | 18 (24.7) | 23 (22.1) | 6 (18.8) | 11 (22.9) | 217 (26.8) | 40 (32.0) | 43 (25.2) | 107 (30.9) | 142 (30.3) | 22 (29.7) | 16 (29.1) | 13 (11.4) | >0.9999 |
| Mercaptopurine | 66 (2.7) | 6 (8.2) | 0 (0.0) | 1 (3.1) | 0 (0.0) | 33 (4.1) | 1 (0.8) | 2 (1.2) | 20 (5.8) | 2 (0.4) | 0 (0.0) | 0 (0.0) | 1 (0.9) | 0.0003 |
| Methotrexate | 80 (3.3) | 2 (2.7) | 0 (0.0) | 1 (3.1) | 1 (2.1) | 43 (5.3) | 7 (5.6) | 7 (4.1) | 5 (1.5) | 10 (2.1) | 2 (2.7) | 0 (0.0) | 2 (1.8) | >0.9999 |
| Adalimumab | 226 (9.3) | 18 (24.7) | 12 (11.5) | 0 (0.0) | 4 (8.3) | 41 (5.1) | 9 (7.2) | 35 (20.5) | 2 (0.6) | 70 (15.0) | 20 (27.0) | 4 (7.3) | 11 (9.7) | <0.0001 |
| Infliximab | 391 (16.2) | 14 (19.2) | 1 (1.0) | 8 (25.0) | 5 (10.4) | 157 (19.4) | 23 (18.4) | 21 (12.3) | 20 (5.8) | 104 (22.2) | 25 (33.8) | 2 (3.6) | 11 (9.7) | <0.0001 |
| Golimumab | 15 (0.6) | 0 (0.0) | 1 (1.0) | 0 (0.0) | 0 (0.0) | 3 (0.4) | 3 (2.4) | 4 (2.3) | 0 (0.0) | 3 (0.6) | 0 (0.0) | 0 (0.0) | 1 (0.9) | >0.9999 |
| Vedolizumab | 100 (4.1) | 11 (15.1) | 2 (1.9) | 1 (3.1) | 2 (4.2) | 20 (2.5) | 6 (4.8) | 13 (7.6) | 8 (2.3) | 26 (5.6) | 0 (0.0) | 4 (7.3) | 7 (6.1) | 0.0009 |
| Ciclosporin | 13 (0.5) | 0 (0.0) | 2 (1.9) | 0 (0.0) | 0 (0.0) | 4 (0.5) | 0 (0.0) | 0 (0.0) | 1 (0.3) | 3 (0.6) | 0 (0.0) | 0 (0.0) | 3 (2.6) | >0.9999 |
| Metronidazole | 87 (3.6) | 2 (2.7) | 6 (5.8) | 1 (3.1) | 0 (0.0) | 32 (4.0) | 1 (0.8) | 3 (1.8) | 12 (3.5) | 10 (2.1) | 15 (20.3) | 1 (1.8) | 4 (3.5) | <0.0001 |
| Beclomethasone | 12 (0.5) | 1 (1.4) | 0 (0.0) | 0 (0.0) | 0 (0.0) | 3 (0.4) | 0 (0.0) | 1 (0.6) | 1 (0.3) | 1 (0.2) | 0 (0.0) | 0 (0.0) | 5 (4.4) | 0.0084 |
| Certolizumab | 4 (0.2) | 0 (0.0) | 0 (0.0) | 0 (0.0) | 0 (0.0) | 2 (0.3) | 0 (0.0) | 0 (0.0) | 1 (0.3) | 0 (0.0) | 1 (1.4) | 0 (0.0) | 0 (0.0) | >0.9999 |
| Ustekinumab | 46 (1.9) | 9 (12.3) | 0 (0.0) | 0 (0.0) | 3 (6.3) | 10 (1.2) | 2 (1.6) | 12 (7.0) | 1 (0.3) | 4 (0.9) | 0 (0.0) | 0 (0.0) | 5 (4.4) | <0.0001 |
| Any biological treatment | 743 (30.7) | 52 (71.2) | 16 (15.4) | 9 (28.1) | 14 (29.2) | 212 (26.2) | 43 (34.4) | 82 (48.0) | 32 (9.3) | 201 (43.0) | 37 (50.0) | 10 (18.2) | 35 (30.7) | <0.0001 |
| *Past surgical treatment, n (%)* |  |  |  |  |  |  |  |  |  |  |  |  |  |  |
| previous year | 144 (5.9) | 6 (8.2) | 3 (2.9) | 1 (3.1) | 5 (10.4) | 48 (5.8) | 10 (8.0) | 6 (3.5) | 17 (4.9) | 27 (5.7) | 5 (6.8) | 5 (8.8) | 11 (9.6) | 0.0001 |
| 1 to 5 years ago | 299 (12.2) | 11 (15.1) | 7 (6.7) | 0 (0.0) | 10 (20.8) | 106 (12.7) | 13 (10.4) | 26 (15.3) | 46 (13.3) | 49 (10.4) | 11 (14.9) | 10 (17.5) | 10 (8.7) |  |
| 5+ years ago | 367 (15.0) | 9 (12.3) | 9 (8.7) | 1 (3.1) | 5 (10.4) | 148 (17.8) | 12 (9.6) | 33 (19.4) | 25 (7.2) | 91 (19.3) | 2 (2.7) | 6 (10.5) | 26 (22.6) |  |
| *Disease activity at last clinical assessment, n (%)* |  |  |  |  |  |  |  |  |  |  |  |  |  |  |
| Remission | 1369 (56.0) | 39 (53.4) | 38 (36.9) | 18 (56.3) | 28 (58.3) | 543 (65.4) | 84 (66.1) | 113 (66.5) | 193 (55.8) | 191 (40.6) | 35 (48.6) | 38 (66.7) | 49 (42.6) | <0.0001 |
| Active disease | 967 (39.6) | 32 (43.8) | 54 (52.4) | 14 (43.8) | 20 (41.7) | 252 (30.4) | 42 (33.1) | 51 (30.0) | 135 (39.0) | 254 (53.9) | 33 (45.8) | 19 (33.3) | 61 (53.0) |  |
| 'don't know'/ ‘don’t remember’ | 108 (4.4) | 2 (2.7) | 11 (10.7) | 0 (0.0) | 0 (0.0) | 35 (4.2) | 1 (0.8) | 6 (3.5) | 18 (5.2) | 26 (5.5) | 4 (5.6) | 0 (0.0) | 5 (4.4) |  |
| *Time of the last assessment of disease activity, n (%)* |  |  |  |  |  |  |  |  |  |  |  |  |  |  |
| previous month | 858 (35.3) | 31 (42.5) | 27 (26.5) | 9 (30.0) | 25 (52.1) | 214 (25.9) | 53 (42.1) | 54 (32.1) | 153 (44.1) | 203 (43.1) | 23 (32.4) | 20 (35.1) | 46 (40.7) | <0.0001 |
| 1-2 months ago | 412 (16.9) | 17 (23.3) | 13 (12.8) | 7 (23.3) | 6 (12.5) | 140 (17.0) | 18 (14.3) | 38 (22.6) | 53 (15.3) | 75 (15.9) | 11 (15.5) | 12 (21.1) | 22 (19.5) |  |
| 2-3 months ago | 401 (16.5) | 13 (17.8) | 18 (17.7) | 6 (20.0) | 10 (20.8) | 129 (15.6) | 22 (17.5) | 50 (29.8) | 50 (14.4) | 70 (14.9) | 13 (18.3) | 6 (10.5) | 14 (12.4) |  |
| 3+ months ago | 761 (31.3) | 12 (16.4) | 44 (43.1) | 8 (26.7) | 7 (14.6) | 343 (41.5) | 33 (26.2) | 26 (15.5) | 91 (26.2) | 123 (26.1) | 24 (33.8) | 19 (33.3) | 31 (27.4) |  |
| *Current disease activity, n (%)* |  |  |  |  |  |  |  |  |  |  |  |  |  |  |
| Remission | 1498 (61.1) | 30 (41.1) | 71 (68.3) | 13 (40.6) | 16 (34.0) | 572 (68.8) | 76 (60.3) | 98 (57.3) | 218 (62.5) | 259 (55.1) | 43 (58.1) | 34 (59.7) | 68 (59.1) | <0.0001 |
| Active disease | 952 (38.9) | 43 (58.9) | 33 (31.7) | 19 (59.4) | 31 (66.0) | 260 (31.3) | 50 (39.7) | 73 (42.7) | 131 (37.5) | 211 (44.9) | 31 (41.9) | 23 (40.4) | 47 (40.9) |  |
| *Current activity of CD or other IBD, n (%)* |  |  |  |  |  |  |  |  |  |  |  |  |  |  |
| Remission | 604 (48.0) | 13 (28.3) | 20 (52.6) | 5 (27.8) | 6 (22.2) | 224 (56.1) | 42 (55.3) | 38 (40.4) | 59 (45.0) | 136 (46.0) | 17 (51.5) | 22 (51.2) | 22 (37.9) | 0.0386 |
| Active disease | 655 (52.0) | 33 (71.7) | 18 (47.4) | 13 (72.2) | 21 (77.8) | 175 (43.9) | 34 (44.7) | 56 (59.6) | 72 (55.0) | 160 (54.1) | 16 (48.5) | 21 (48.8) | 36 (62.1) |  |
| P-HBI score, mean (SD) | 5.65 (5.00) | 7.30 (5.60) | 4.63 (3.56) | 6.72 (4.43) | 7.33 (5.11) | 5.06 (4.93) | 4.64 (3.45) | 7.00 (6.14) | 5.89 (4.63) | 5.56 (4.93) | 6.18 (5.82) | 5.07 (4.90) | 7.09 (5.24) | 0.0217 |
| *Current activity of UC, n (%)* |  |  |  |  |  |  |  |  |  |  |  |  |  |  |
| Remission | 894 (75.1) | 17 (63.0) | 51 (77.3) | 8 (57.1) | 10 (50.0) | 348 (80.4) | 34 (68.0) | 60 (77.9) | 159 (72.9) | 123 (70.7) | 26 (63.4) | 12 (85.7) | 46 (80.7) | >0.9999 |
| Active disease | 297 (24.9) | 10 (37.0) | 15 (22.7) | 6 (42.9) | 10 (50.0) | 85 (19.6) | 16 (32.0) | 17 (22.1) | 59 (27.1) | 51 (29.3) | 15 (36.6) | 2 (14.3) | 11 (19.3) |  |
| P-SCCAI score (UC), mean (SD) | 3.64 (3.14) | 4.33 (3.51) | 3.21 (3.07) | 4.86 (3.82) | 5.45 (4.12) | 3.25 (2.94) | 4.06 (3.15) | 3.68 (2.90) | 3.91 (3.21) | 3.75 (3.06) | 3.80 (3.30) | 3.07 (4.05) | 4.07 (3.45) | >0.9999 |
| UC patients with stoma, n (%) | 87 (7.3) | 0 (0.0) | 0 (0.0) | 1 (7.1) | 1 (4.8) | 38 (8.8) | 8 (15.7) | 9 (11.7) | 8 (3.7) | 10 (5.8) | 3 (7.3) | 2 (14.3) | 7 (12.3) | >0.9999 |
| Penetrating CD course, n (%) | 376 (30.7) | 9 (20.0) | 8 (22.9) | 5 (29.4) | 11 (42.3) | 139 (35.2) | 16 (22.5) | 15 (16.7) | 32 (25.0) | 100 (34.1) | 8 (26.7) | 13 (31.0) | 20 (37.0) | >0.9999 |
| Retired, n (%) | 29 (1.2) | 0 (0.0) | 3 (2.9) | 0 (0.0) | 0 (0.0) | 9 (1.1) | 1 (0.8) | 1 (0.6) | 4 (1.2) | 5 (1.1) | 2 (2.7) | 1 (1.8) | 3 (2.6) | >0.9999 |
| On a disability pension, n (%) | 140 (5.7) | 0 (0.0) | 15 (14.4) | 0 (0.0) | 12 (25.0) | 86 (10.3) | 3 (2.4) | 2 (1.2) | 13 (3.7) | 2 (0.4) | 1 (1.4) | 4 (7.0) | 2 (1.7) | <0.0001 |
| With disability certificate | 330 (13.4) | 1 (1.4) | 6 (5.8) | 1 (3.1) | 6 (12.5) | 12 (1.4) | 31 (24.4) | 56 (32.8) | 108 (31.0) | 60 (12.7) | 1 (1.4) | 3 (5.3) | 45 (39.1) | <0.0001 |
| Student, n (%) | 90 (3.7) | 0 (0.0) | 2 (1.9) | 0 (0.0) | 2 (4.2) | 40 (4.8) | 4 (3.2) | 3 (1.8) | 13 (3.7) | 13 (2.8) | 6 (8.1) | 3 (5.3) | 4 (3.5) | >0.9999 |
| Number of consultations with clinician in previous month, mean (SD) | 1.67 (3.09) | 2.25 (2.55) | 1.93 (3.83) | 1.28 (1.51) | 1.90 (2.50) | 1.04 (2.56) | 1.68 (1.66) | 5.29 (5.30) | 1.46 (2.53) | 1.37 (2.34) | 2.32 (4.25) | 1.70 (2.58) | 1.68 (3.34) | <0.0001 |
| Length of hospital stay in previous month, mean (SD) | 0.72 (2.35) | 0.90 (1.96) | 1.04 (2.34) | 1.07 (4.59) | 0.40 (1.66) | 0.57 (1.44) | 0.43 (1.77) | 0.44 (2.78) | 0.50 (1.82) | 1.18 (3.32) | 1.60 (4.23) | 0.18 (0.60) | 0.65 (2.26) | <0.0001 |

## Supplementary Table 4. Fractional logit models for impairment scores (WPAI).

|  | Regular activity, OR (95% CI) | Absenteeism, OR (95% CI) | Presenteeism, OR (95% CI) |
| --- | --- | --- | --- |
| a year increase in age | 0.9900 (0.9809 - 0.9993) | 0.9766 (0.9471 - 1.0070) | 0.9919 (0.9782 - 1.0059) |
| sex: men vs women | 0.8315 (0.6980 - 0.9905) | 0.8919 (0.5611 - 1.4177) | 0.9824 (0.7794 - 1.2383) |
| place: city <100K vs city ≥100K | 1.0064 (0.8345 - 1.2137) | 0.8946 (0.5405 - 1.4808) | 0.9835 (0.7630 - 1.2677) |
| place: village vs city ≥100K | 0.9948 (0.8008 - 1.2357) | 1.0214 (0.5560 - 1.8765) | 0.8889 (0.6706 - 1.1784) |
| a year increase in age at diagnosis | 1.0008 (0.9914 - 1.0103) | 1.0197 (0.9886 - 1.0517) | 0.9992 (0.9854 - 1.0132) |
| Comorbidity: any vs no | 1.3053 (1.0991 - 1.5502) | 1.0914 (0.7038 - 1.6924) | 1.3053 (1.0423 - 1.6348) |
| surgery: previous year vs no surgery | 1.4766 (1.0911 - 1.9983) | 2.2850 (1.0527 - 4.9598) | 1.1122 (0.6869 - 1.8007) |
| surgery: 1 to 5 years ago vs no surgery | 1.0770 (0.8370 - 1.3856) | 1.4179 (0.7576 - 2.6538) | 0.9087 (0.6565 - 1.2577) |
| surgery: 5+ years ago vs no surgery | 0.9539 (0.7353 - 1.2374) | 1.2659 (0.5923 - 2.7059) | 0.8523 (0.5994 - 1.2118) |
| biological treatment: currently vs no | 1.2096 (1.0071 - 1.4529) | 1.5331 (0.9281 - 2.5327) | 1.1700 (0.9120 - 1.5009) |
| UC vs CD or other IBD | 1.3808 (1.1470 - 1.6623) | 4.9366 (3.0957 - 7.8720) | 2.8290 (2.2576 - 3.5451) |
| active disease vs remission | 3.6961 (3.1199 - 4.3787) | 1.5372 (0.9471 - 2.4952) | 1.2511 (0.9818 - 1.5943) |
| Bulgaria vs Belgium | 0.8872 (0.3148 - 2.5002) | 1.5947 (0.3191 - 7.9683) | 0.7596 (0.3621 - 1.5935) |
| Cyprus vs Belgium | 1.0484 (0.2829 - 3.8848) | 0.9194 (0.1587 - 5.3265) | 0.6322 (0.2441 - 1.6368) |
| Czech Republic vs Belgium | 0.7172 (0.2592 - 1.9845) | 2.0268 (0.4000 - 10.2685) | 0.4650 (0.1858 - 1.1639) |
| Denmark vs Belgium | 0.5602 (0.3134 - 1.0012) | 1.1587 (0.3678 - 3.6502) | 0.7241 (0.4330 - 1.2110) |
| Greece vs Belgium | 0.6026 (0.3050 - 1.1907) | 1.0803 (0.2630 - 4.4366) | 0.9525 (0.4829 - 1.8787) |
| Spain vs Belgium | 0.6020 (0.2899 - 1.2503) | 2.8134 (0.7800 - 10.1473) | 0.6469 (0.3277 - 1.2774) |
| Poland vs Belgium | 0.7520 (0.3825 - 1.4786) | 1.4749 (0.4198 - 5.1824) | 1.0080 (0.5756 - 1.7652) |
| Portugal vs Belgium | 0.6300 (0.3361 - 1.1808) | 1.6260 (0.5078 - 5.2068) | 0.8394 (0.4969 - 1.4180) |
| Romania vs Belgium | 0.7233 (0.3186 - 1.6421) | 2.1400 (0.4676 - 9.7936) | 1.0809 (0.5288 - 2.2094) |
| Hungary vs Belgium | 0.8203 (0.2850 - 2.3610) | 1.1711 (0.1917 - 7.1545) | 0.9407 (0.4474 - 1.9776) |
| Italy vs Belgium | 0.6686 (0.3194 - 1.3993) | 3.1385 (0.8492 - 11.5997) | 1.0235 (0.4864 - 2.1535) |
| Occupational activity: yes vs no | 0.4995 (0.2475 - 1.0079) | - | - |
| Bulgaria#Employed vs Belgium#NotEmployed | 0.9951 (0.2844 - 3.4816) | - | - |
| Cyprus#Employed vs Belgium#NotEmployed | 0.7169 (0.1330 - 3.8649) | - | - |
| Czech Republic#Employed vs Belgium#NotEmployed | 0.8385 (0.2320 - 3.0299) | - | - |
| Denmark#Employed vs Belgium#NotEmployed | 1.3635 (0.6362 - 2.9221) | - | - |
| Greece#Employed vs Belgium#NotEmployed | 1.6917 (0.6580 - 4.3493) | - | - |
| Spain#Employed vs Belgium#NotEmployed | 1.6970 (0.6615 - 4.3535) | - | - |
| Poland#Employed vs Belgium#NotEmployed | 1.3659 (0.5816 - 3.2075) | - | - |
| Portugal#Employed vs Belgium#NotEmployed | 1.2790 (0.5672 - 2.8838) | - | - |
| Romania#Employed vs Belgium#NotEmployed | 1.6279 (0.5545 - 4.7790) | - | - |
| Hungary#Employed vs Belgium#NotEmployed | 1.0821 (0.2876 - 4.0717) | - | - |
| Italy#Employed vs Belgium#NotEmployed | 2.0828 (0.7781 - 5.5753) | - | - |
| _cons | 0.5971 (0.3208 - 1.1114) | 0.0311 (0.0064 - 0.1516) | 0.2668 (0.1361 - 0.5229) |
| Pseudo R2 | 0.0947 | 0.1148 | 0.0547 |
| Log pseudolikelihood | -1833.89 | -623.978 | -1056.6 |

## Supplementary Table 5. Two-part models for indirect costs.

|  | Informal care (95% CI), N= 3,038 | Absenteeism (95% CI), N= 2,082 | Presenteeism (95% CI), N= 2,005 |
| --- | --- | --- | --- |
| **logit part (ORs are presented)** |  |  |  |
| a year increase in age | 0.9785 (0.9602 - 0.9971) | 0.9815 (0.9564 - 1.0073) | 0.9871 (0.9640 - 1.0106) |
| sex: men vs women | 1.0291 (0.7186 - 1.4739) | 0.9469 (0.6256 - 1.4333) | 1.1309 (0.7591 - 1.6849) |
| place: city <100K vs city ≥100K | 1.0500 (0.7138 - 1.5444) | 1.0107 (0.6462 - 1.5808) | 0.9743 (0.6260 - 1.5165) |
| place: village vs city ≥100K | 0.8691 (0.5493 - 1.3750) | 0.8266 (0.4813 - 1.4196) | 0.8517 (0.5220 - 1.3895) |
| a year increase in age at diagnosis | 0.9931 (0.9732 - 1.0134) | 1.0164 (0.9911 - 1.0423) | 1.0040 (0.9816 - 1.0269) |
| Comorbidity: any vs no | 1.4867 (1.0405 - 2.1243) | 1.2098 (0.8094 - 1.8082) | 1.5712 (1.0739 - 2.2987) |
| surgery: previous year vs no surgery | 1.5589 (0.8480 - 2.8659) | 1.5310 (0.6977 - 3.3596) | 0.9218 (0.3912 - 2.1721) |
| surgery: 1 to 5 years ago vs no surgery | 1.0041 (0.5954 - 1.6932) | 1.0587 (0.5802 - 1.9319) | 0.9637 (0.5250 - 1.7693) |
| surgery: 5+ years ago vs no surgery | 0.9078 (0.5243 - 1.5720) | 1.0112 (0.5287 - 1.9340) | 1.0305 (0.5689 - 1.8667) |
| Occupational activity: yes vs no | 0.4568 (0.3214 - 0.6492) | - | - |
| biological treatment: currently vs no | 1.0719 (0.7296 - 1.5748) | 1.4478 (0.9190 - 2.2810) | 1.0788 (0.7062 - 1.6479) |
| UC vs CD or other IBD | 1.0230 (0.6914 - 1.5136) | 1.6369 (1.0428 - 2.5696) | 1.1821 (0.7568 - 1.8464) |
| active disease vs remission | 3.7312 (2.6259 - 5.3018) | 4.8264 (3.1803 - 7.3246) | 3.3205 (2.1632 - 5.0972) |
| Bulgaria vs Belgium | 1.2962 (0.4000 - 4.2002) | 0.9339 (0.2061 - 4.2320) | 0.3886 (0.0795 - 1.8993) |
| Cyprus vs Belgium | 0.3871 (0.0685 - 2.1883) | 0.9508 (0.1302 - 6.9424) | 0.7102 (0.0807 - 6.2533) |
| Czech Republic vs Belgium | 0.7550 (0.1782 - 3.1987) | 1.1568 (0.2186 - 6.1219) | 0.2422 (0.0384 - 1.5288) |
| Denmark vs Belgium | 0.3925 (0.1585 - 0.9716) | 0.4963 (0.1525 - 1.6147) | 0.4305 (0.1180 - 1.5697) |
| Greece vs Belgium | 1.0334 (0.3668 - 2.9111) | 1.3614 (0.3314 - 5.5924) | 0.5291 (0.1140 - 2.4548) |
| Spain vs Belgium | 0.5798 (0.2040 - 1.6480) | 1.1420 (0.3154 - 4.1348) | 0.2737 (0.0641 - 1.1690) |
| Poland vs Belgium | 0.7001 (0.2642 - 1.8549) | 0.7283 (0.2097 - 2.5299) | 0.7243 (0.1811 - 2.8965) |
| Portugal vs Belgium | 0.5339 (0.2092 - 1.3628) | 1.0763 (0.3273 - 3.5396) | 0.4115 (0.1105 - 1.5331) |
| Romania vs Belgium | 2.0642 (0.6629 - 6.4278) | 1.3939 (0.2929 - 6.6329) | 0.6456 (0.1185 - 3.5157) |
| Hungary vs Belgium | 0.4590 (0.1055 - 1.9978) | 0.8832 (0.1599 - 4.8791) | 0.6954 (0.1221 - 3.9618) |
| Italy vs Belgium | 0.9269 (0.3149 - 2.7286) | 1.3408 (0.3380 - 5.3187) | 0.4079 (0.0861 - 1.9318) |
| _cons | 1.2452 (0.3939 - 3.9365) | 0.2518 (0.0589 - 1.0768) | 3.0225 (0.6559 - 13.9292) |
| **Generalized linear model part** |  |  |  |
| a year increase in age | -0.0179 (-0.0385 - 0.0027) | -0.0145 (-0.0384 - 0.0093) | 0.0002 (-0.0104 - 0.0109) |
| sex: men vs women | 0.0540 (-0.3416 - 0.4496) | 0.0524 (-0.4636 - 0.5684) | 0.0208 (-0.1468 - 0.1884) |
| place: city <100K vs city ≥100K | 0.0550 (-0.3933 - 0.5032) | -0.2430 (-0.6667 - 0.1806) | 0.0395 (-0.1495 - 0.2285) |
| place: village vs city ≥100K | 0.1886 (-0.3021 - 0.6794) | 0.1937 (-0.3435 - 0.7308) | -0.0087 (-0.2256 - 0.2081) |
| a year increase in age at diagnosis | 0.0060 (-0.0153 - 0.0273) | 0.0098 (-0.0111 - 0.0307) | -0.0001 (-0.0100 - 0.0099) |
| Comorbidity: any vs no | 0.1027 (-0.2961 - 0.5015) | 0.1522 (-0.2778 - 0.5822) | -0.0064 (-0.1621 - 0.1494) |
| surgery: previous year vs no surgery | 0.0680 (-0.4519 - 0.5880) | 0.5262 (-0.1679 - 1.2202) | 0.0857 (-0.3153 - 0.4868) |
| surgery: 1 to 5 years ago vs no surgery | 0.1427 (-0.4409 - 0.7264) | 0.1972 (-0.4265 - 0.8210) | -0.1143 (-0.3322 - 0.1036) |
| surgery: 5+ years ago vs no surgery | 0.1125 (-0.5738 - 0.7988) | 0.0744 (-0.5226 - 0.6714) | -0.0733 (-0.3777 - 0.2311) |
| Occupational activity: yes vs no | -0.3143 (-0.6819 - 0.0534) | - | - |
| biological treatment: currently vs no | -0.0182 (-0.4001 - 0.3636) | -0.0082 (-0.4953 - 0.4789) | 0.1718 (0.0061 - 0.3376) |
| UC vs CD or other IBD | -0.1401 (-0.5527 - 0.2725) | 0.1157 (-0.2702 - 0.5015) | 0.0697 (-0.1151 - 0.2545) |
| active disease vs remission | 0.5754 (0.1782 - 0.9726) | 0.4377 (0.0063 - 0.8690) | 0.3970 (0.2413 - 0.5527) |
| Bulgaria vs Belgium | -0.0717 (-1.1705 - 1.0271) | -1.1015 (-2.3277 - 0.1246) | -1.4881 (-1.9474 - -1.0288) |
| Cyprus vs Belgium | 0.6228 (-1.0370 - 2.2827) | -0.6318 (-1.9291 - 0.6655) | -1.0560 (-1.6427 - -0.4693) |
| Czech Republic vs Belgium | -0.7948 (-1.7111 - 0.1216) | -0.1603 (-1.5296 - 1.2089) | -1.1683 (-1.7126 - -0.6241) |
| Denmark vs Belgium | 0.3352 (-0.4125 - 1.0828) | 0.7499 (-0.1219 - 1.6217) | -0.0225 (-0.3406 - 0.2955) |
| Greece vs Belgium | -0.5152 (-1.3474 - 0.3171) | -1.2315 (-2.3050 - -0.1580) | -0.9860 (-1.4131 - -0.5590) |
| Spain vs Belgium | 0.5431 (-0.4240 - 1.5102) | 0.4757 (-0.5284 - 1.4799) | -0.5570 (-0.9601 - -0.1540) |
| Poland vs Belgium | -0.8807 (-1.6382 - -0.1233) | -0.5240 (-1.7517 - 0.7036) | -1.1238 (-1.4977 - -0.7499) |
| Portugal vs Belgium | -0.3985 (-1.1500 - 0.3529) | -0.5243 (-1.4279 - 0.3793) | -0.7873 (-1.1642 - -0.4104) |
| Romania vs Belgium | -0.3797 (-1.2234 - 0.4640) | -0.7612 (-1.8359 - 0.3135) | -1.3123 (-1.7959 - -0.8286) |
| Hungary vs Belgium | -0.2560 (-2.9125 - 2.4005) | 0.5452 (-2.3925 - 3.4830) | -1.2276 (-1.6475 - -0.8077) |
| Italy vs Belgium | 0.7918 (-0.1457 - 1.7293) | 0.3351 (-0.6779 - 1.3482) | -0.3611 (-0.7721 - 0.0500) |
| _cons | 9.7647 (8.8195 - 10.7100) | 9.5522 (8.1967 - 10.9078) | 9.8125 (9.2837 - 10.3412) |
| **Pseudo R2 (logit)** | 0.159 | 0.1403 | 0.0817 |
| **Log pseudolikelihood (two-part model)** | -10501.8 | -8277.68 | -14918.1 |
| **Mean squared error** | 2.76e+08 | 4.67e+08 | 1.56e+08 |
| **Mean absolute error** | 4,001.655 | 8,086.851 | 8,178.554 |

## Supplementary Table 6. Raw mean (SD; N) of indirect costs by country, disease activity and IBD type (in 2019 €).

|  | | Informal care | | Absenteeism | | Presenteeism | |
| --- | --- | --- | --- | --- | --- | --- | --- |
|  |  | Remission | Active | Remission | Active | Remission | Active |
| CD | BE | 963 (1,830.24; 13) | 4,902 (8,469.93; 31) | 7,913 (21,803.06; 13) | 18,703 (31,263.92; 33) | 14,873 (12,621.82; 13) | 26,414 (17,325.42; 33) |
|  | BG | 544 (1,298.91; 18) | 6,972 (14,886.06; 17) | 623 (1,419.68; 19) | 2,310 (3,155.87; 16) | 1,864 (1,848.97; 18) | 5,974 (4,265.06; 15) |
|  | CY | 0 (0.00; 5) | 13,178 (29,969.79; 11) | 1,549 (3,464.63; 5) | 2,744 (3,475.28; 12) | 3,293 (4,521.48; 5) | 10,015 (6,584.36; 12) |
|  | CZ | 2,138 (3,311.95; 6) | 3,000 (6,739.79; 19) | 315 (705.04; 5) | 11,075 (24,904.00; 20) | 552 (1,233.82; 5) | 5,908 (6,737.10; 21) |
|  | DK | 1,094 (8,583.65; 220) | 5,364 (16,232.74; 167) | 2,938 (14,814.64; 220) | 12,067 (24,343.79; 173) | 9,266 (12,640.27; 220) | 21,333 (19,624.83; 167) |
|  | GR | 1,565 (5,866.79; 37) | 3,331 (8,823.94; 32) | 479 (1,365.90; 41) | 2,108 (4,805.31; 32) | 2,991 (4,606.05; 40) | 7,330 (5,843.55; 31) |
|  | ES | 650 (3,779.60; 37) | 22,267 (43,242.64; 50) | 486 (1,729.46; 38) | 20,063 (39,420.98; 56) | 3,989 (5,656.03; 36) | 11,715 (12,288.66; 51) |
|  | PL | 396 (1,126.73; 57) | 2,373 (3,868.95; 66) | 1,390 (8,154.92; 58) | 8,584 (37,126.59; 70) | 3,372 (3,942.23; 54) | 7,234 (8,005.15; 68) |
|  | PT | 981 (5,312.72; 129) | 2,870 (7,919.40; 150) | 2,535 (8,320.38; 134) | 6,244 (13,384.63; 153) | 4,299 (6,171.67; 128) | 11,739 (17,118.58; 147) |
|  | RO | 3,296 (6,544.45; 15) | 11,190 (23,816.95; 14) | 4,153 (7,596.09; 16) | 6,046 (8,465.42; 15) | 4,429 (4,769.75; 15) | 4,964 (3,439.46; 14) |
|  | HU | 165 (538.40; 21) | 12,578 (43,424.95; 16) | 222 (581.44; 22) | 33,308 (132,149.61; 21) | 2,883 (2,947.42; 22) | 7,492 (5,386.03; 21) |
|  | IT | 1,735 (6,534.97; 22) | 12,900 (22,733.54; 36) | 3,170 (10,806.28; 22) | 17,552 (33,286.31; 36) | 7,094 (8,921.51; 21) | 13,619 (13,150.45; 30) |
| UC | BE | 3,620 (10,670.40; 15) | 5,634 (10,379.87; 10) | 2,755 (5,773.27; 16) | 4,829 (10,661.19; 10) | 9,815 (10,223.89; 16) | 35,060 (25,845.87; 10) |
|  | BG | 2,433 (10,538.69; 46) | 4,019 (6,906.68; 14) | 1,026 (3,296.36; 50) | 4,224 (7,381.25; 13) | 2,770 (3,586.78; 48) | 4,311 (5,073.16; 15) |
|  | CY | 129 (315.05; 6) | 0 (0.00; 6) | 3,268 (5,251.76; 8) | 5,003 (7,995.37; 6) | 9,144 (6,923.82; 7) | 7,779 (4,885.04; 6) |
|  | CZ | 683 (1,197.83; 7) | 3,976 (5,155.74; 10) | 709 (1,596.11; 10) | 17,736 (38,495.43; 10) | 3,311 (3,909.05; 9) | 11,107 (7,984.64; 10) |
|  | DK | 564 (2,672.84; 339) | 3,794 (13,276.99; 81) | 2,986 (13,286.34; 344) | 15,300 (29,859.68; 84) | 11,042 (15,676.11; 332) | 25,136 (23,796.12; 83) |
|  | GR | 2,268 (6,938.10; 31) | 7,009 (15,951.47; 14) | 3,016 (6,683.64; 33) | 5,195 (5,407.99; 15) | 6,429 (7,236.91; 32) | 9,675 (8,869.82; 16) |
|  | ES | 350 (1,363.73; 58) | 2,922 (6,053.07; 15) | 5,086 (13,873.61; 60) | 16,866 (22,025.47; 17) | 7,455 (10,999.70; 55) | 12,139 (10,206.16; 17) |
|  | PL | 827 (3,183.15; 151) | 2,176 (3,135.63; 57) | 1,234 (4,910.38; 154) | 4,361 (7,320.42; 58) | 5,533 (6,437.42; 151) | 8,021 (6,812.61; 56) |
|  | PT | 1,089 (4,582.17; 120) | 3,784 (8,257.18; 46) | 2,384 (8,113.08; 120) | 6,914 (10,989.49; 48) | 4,455 (6,033.29; 114) | 10,954 (11,407.64; 47) |
|  | RO | 2,371 (4,568.65; 19) | 5,719 (8,834.24; 15) | 727 (1,170.59; 24) | 4,462 (7,858.92; 15) | 4,805 (5,339.41; 24) | 5,546 (4,457.90; 14) |
|  | HU | 0 (0.00; 11) | 2,597 (2,448.21; 2) | 762 (1,785.04; 12) | 1,525 (2,156.05; 2) | 4,208 (3,038.01; 12) | 8,842 (431.21; 2) |
|  | IT | 2,989 (9,234.65; 41) | 37,814 (74,390.44; 11) | 1,966 (4,465.23; 44) | 32,082 (25,878.75; 11) | 9,037 (10,139.73; 37) | 12,059 (16,223.08; 11) |

## Supplementary Table 7. Adjusted mean indirect costs (SE) by country and IBD type (in 2019 €).

| Country | IBD | Informal care, per patient | Absenteeism, per working patient | Presenteeism, per working patient | Absenteeism, per patient (67% employed) | Presenteeism, per patient (67% employed) | All indirect costs, per patient (67% employed) |
| --- | --- | --- | --- | --- | --- | --- | --- |
| BE | CD | 5,665 (1,100) | 5,831 (1,550) | 18,001 (1,817) | 3,909 (1,039) | 12,068 (1,218) | 21,642 (1,942) |
|  | UC | 4,977 (1,056) | 6,921 (1,840) | 21,664 (2,191) | 4,640 (1,233) | 14,524 (1,469) | 24,140 (2,189) |
| BG | CD | 5,917 (1,586) | 1,869 (549) | 3,205 (413) | 1,253 (368) | 2,149 (277) | 9,319 (1,652) |
|  | UC | 5,192 (1,412) | 2,229 (654) | 3,900 (496) | 1,495 (438) | 2,615 (332) | 9,302 (1,516) |
| CY | CD | 6,243 (3,052) | 3,019 (1,096) | 5,838 (1,029) | 2,024 (734) | 3,914 (690) | 12,181 (3,214) |
|  | UC | 5,506 (2,794) | 3,596 (1,306) | 7,051 (1,241) | 2,411 (876) | 4,727 (832) | 12,644 (3,044) |
| CZ | CD | 2,228 (554) | 5,347 (1,863) | 3,699 (731) | 3,585 (1,249) | 2,480 (490) | 8,293 (1,452) |
|  | UC | 1,960 (500) | 6,285 (2,145) | 4,532 (881) | 4,213 (1,438) | 3,038 (591) | 9,211 (1,633) |
| DK | CD | 4,724 (750) | 8,183 (1,257) | 14,340 (730) | 5,486 (842) | 9,614 (489) | 19,824 (1,230) |
|  | UC | 4,166 (653) | 10,203 (1,431) | 17,427 (929) | 6,840 (959) | 11,683 (623) | 22,689 (1,317) |
| ES | CD | 7,357 (1,674) | 10,037 (2,094) | 7,164 (814) | 6,729 (1,404) | 4,803 (546) | 18,889 (2,252) |
|  | UC | 6,478 (1,566) | 11,807 (2,265) | 8,761 (973) | 7,915 (1,518) | 5,874 (652) | 20,267 (2,277) |
| GR | CD | 3,436 (606) | 1,980 (455) | 5,813 (634) | 1,327 (305) | 3,897 (425) | 8,661 (800) |
|  | UC | 3,018 (582) | 2,302 (518) | 7,045 (774) | 1,543 (347) | 4,723 (519) | 9,285 (854) |
| HU | CD | 2,884 (2,088) | 9,416 (7,449) | 4,894 (537) | 6,313 (4,994) | 3,281 (360) | 12,477 (5,425) |
|  | UC | 2,542 (1,854) | 11,273 (8,872) | 5,912 (675) | 7,557 (5,948) | 3,963 (452) | 14,062 (6,246) |
| IT | CD | 12,063 (2,630) | 9,419 (1,931) | 10,050 (1,141) | 6,314 (1,294) | 6,737 (765) | 25,115 (3,029) |
|  | UC | 10,601 (2,414) | 10,963 (2,172) | 12,221 (1,376) | 7,349 (1,456) | 8,193 (923) | 26,143 (2,966) |
| PL | CD | 1,965 (281) | 2,900 (825) | 5,480 (408) | 1,944 (553) | 3,674 (274) | 7,583 (678) |
|  | UC | 1,729 (259) | 3,519 (819) | 6,617 (467) | 2,359 (549) | 4,436 (313) | 8,524 (683) |
| PT | CD | 2,737 (385) | 3,585 (479) | 6,581 (494) | 2,403 (321) | 4,412 (331) | 9,552 (601) |
|  | UC | 2,410 (388) | 4,233 (624) | 8,002 (638) | 2,838 (418) | 5,364 (428) | 10,613 (713) |
| RO | CD | 5,191 (946) | 3,204 (763) | 4,417 (582) | 2,148 (512) | 2,961 (390) | 10,300 (1,144) |
|  | UC | 4,548 (836) | 3,719 (924) | 5,341 (695) | 2,494 (619) | 3,580 (466) | 10,622 (1,140) |
